# Supplementary material for: An instantly fixable and self-adaptive scaffold for skull regeneration by autologous stem cell recruitment and angiogenesis
Source: Nat Commun. 2022 May 6;13:2499. doi: 10.1038/s41467-022-30243-5 (PMC9076642; doi:10.1038/s41467-022-30243-5)
Supplement: Supplementary file 1 — Supplementary Information file [file 41467_2022_30243_MOESM1_ESM.pdf]

**PAn Instantly Fixable and Self-adaptive Scaffold for Skull Regeneration by Autologous Stem Cell Recruitment and Angiogenesis**

Gonggong Lu[1,2,3†], Yang Xu[1,3†], Quanying Liu[1,3], Manyu Chen[1,3], Huan Sun[1,3], Peilei Wang[1,3], Xing Li[1,3], Yuxiang Wang[1,3], Xiang Li[2], Xuhui Hui[2], En Luo[4], Jun Liu[5], Qing Jiang[1,3], Jie Liang[1,3], Yujiang Fan[1,3*], Yong Sun[1,3*], Xingdong Zhang[1,3]

[1]National Engineering Research Center for Biomaterials, Sichuan University, 29# Wangjiang Road, Chengdu, Sichuan, 610064, P. R. China.

[2]Department of Neurosurgery, West China Hospital, Sichuan University, 37# Guoxue Lane, Chengdu, Sichuan, 610041, P. R. China.

[3]College of Biomedical Engineering, Sichuan University, 29# Wangjiang Road, Chengdu, Sichuan, 610064, P. R. China.

[4]State Key Laboratory of Oral Diseases & National Clinical Research Center for Oral Diseases & Department of Oral and Maxillofacial Surgery, West China Hospital of Stomatology, Sichuan University, 14#, 3rd, Section of Renmin South Road, Chengdu, Sichuan, 610041, P.R. China.

[5]School of Biological Science & Medical Engineering, Southeast University, 2# Sipai Building, Xuanwu District, Nanjing, Jiangsu, 210096, P. R. China.

†These authors contributed equally to this work.

*Corresponding author.

Email: fan_yujiang@scu.edu.cn (Y.F.); sunyong8702@scu.edu.cn (Y.S.)

*Supplementary Information*

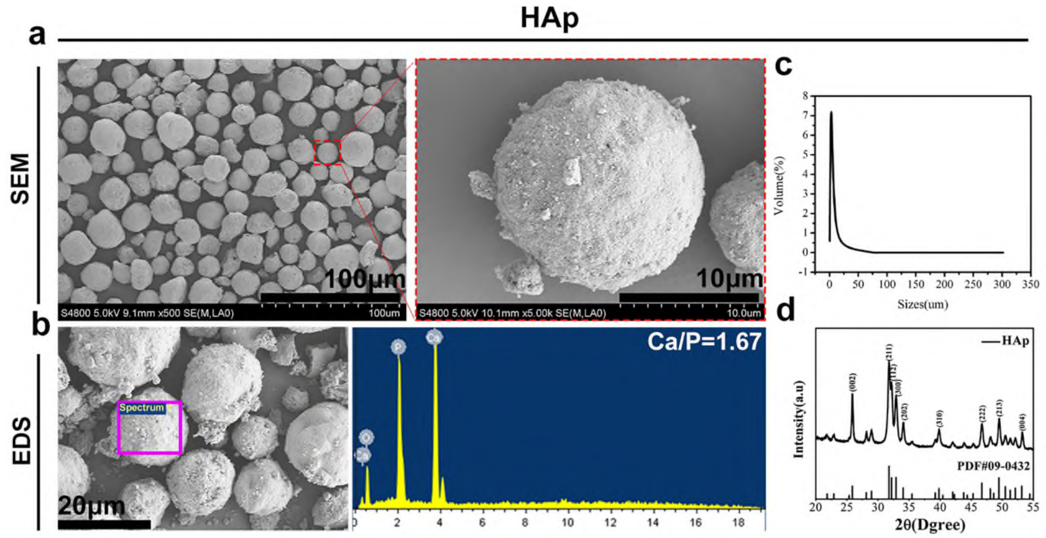


**Supplementary Figure 1.** Characterization of micron hydroxyapatite. a) SEM images of μHAp. b) EDS analysis of μHAp. c) Particle size distribution of μHAp. d) XRD spectrum of μHAp.

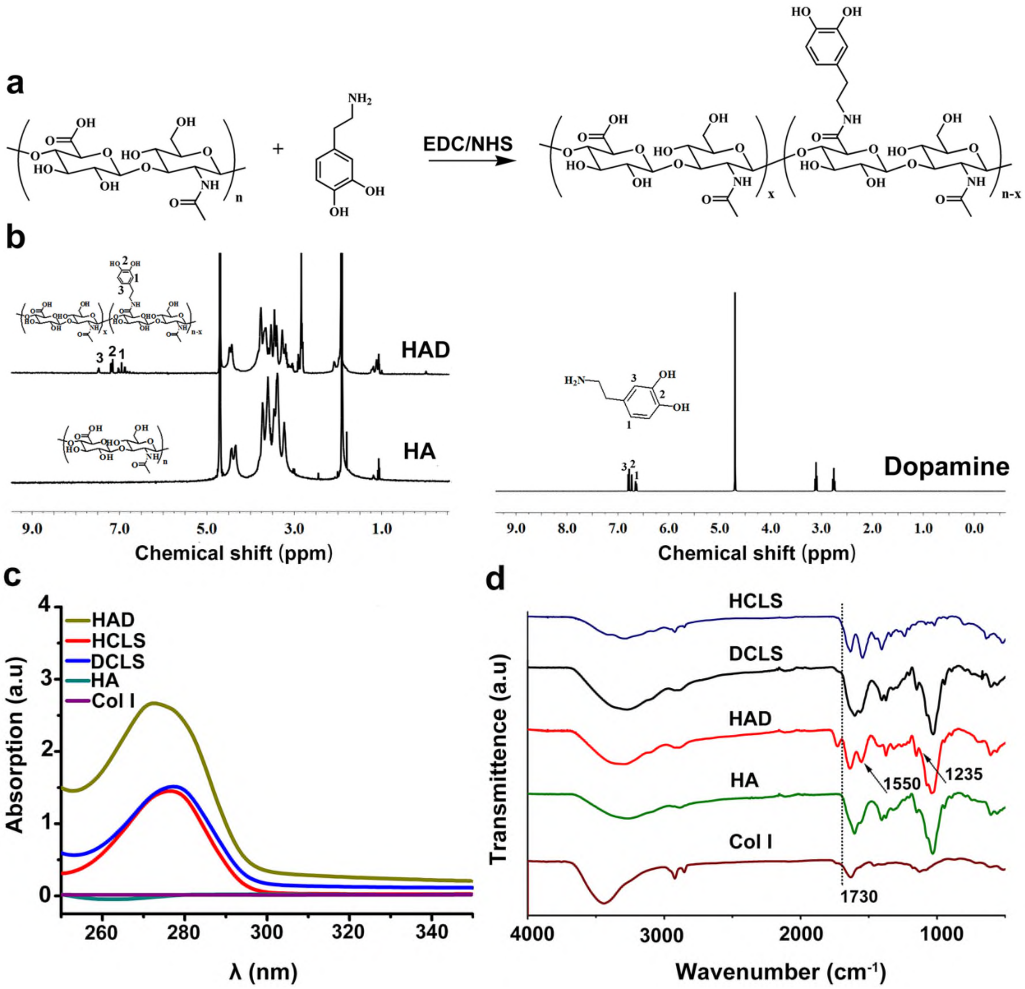


**Supplementary Figure 2.** Synthesis and characterization of HAD. a) Synthetic route of HAD based on amidation. b) $^1$H NMR ($D_2O$) spectra of HA and its derivatives (HAD). c) UV absorption spectrum of various specimens. d) FTIR spectrum of various samples.

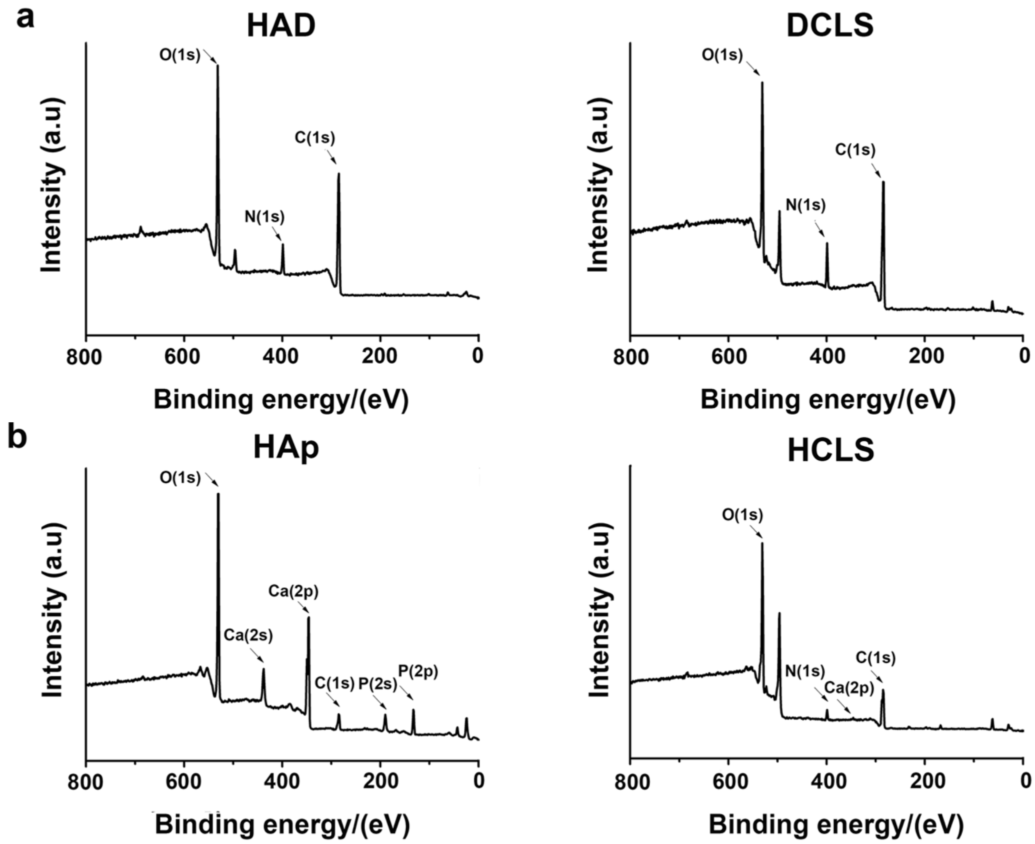


**Supplementary Figure 3.** Full x-ray photoelectron spectroscopy spectra. a) Chemical bonding between HAD and Col I based on x-ray photoelectron spectroscopy. b) Calcium chelation analysis in HCLS based on x-ray photoelectron spectroscopy.

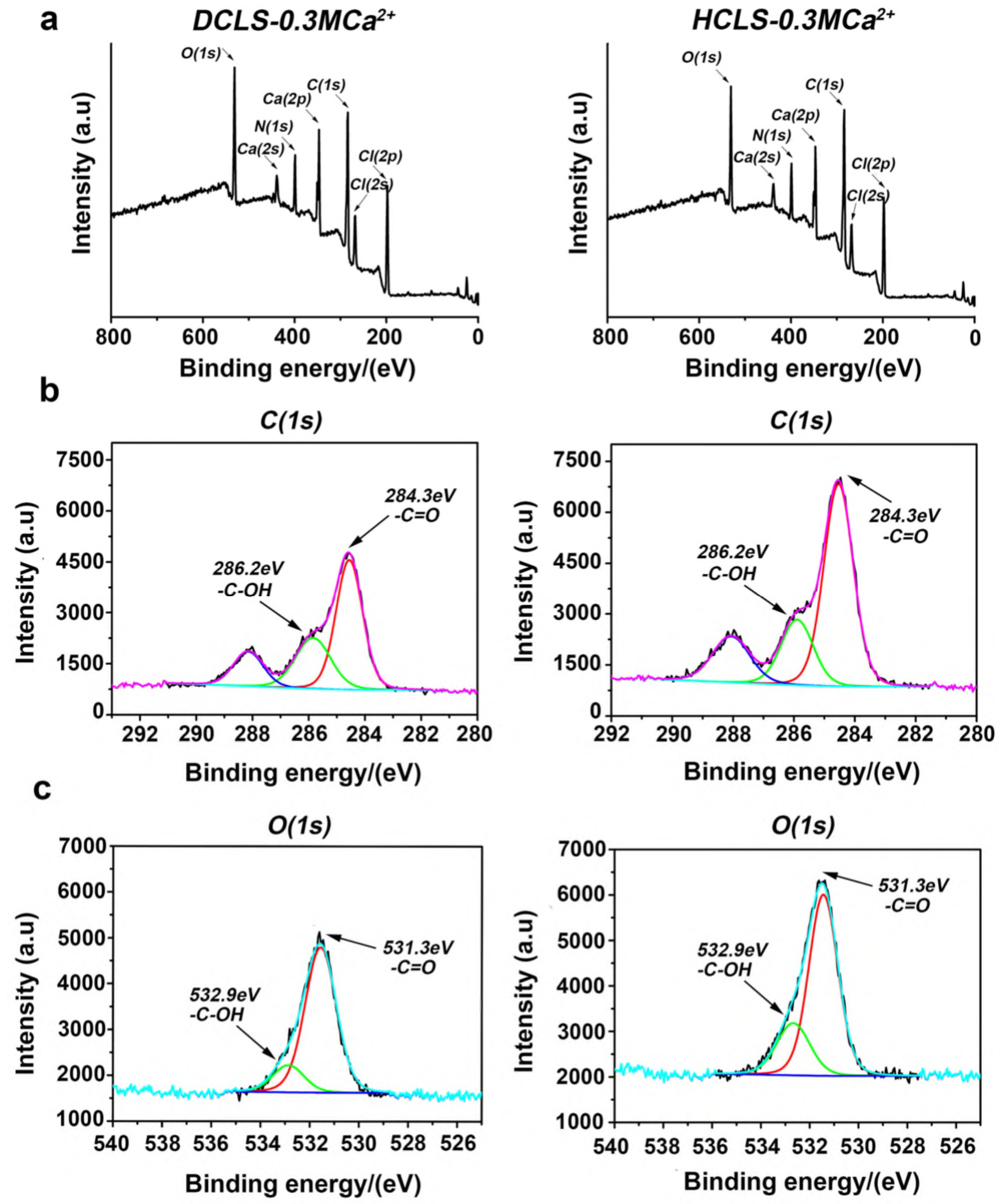


**Supplementary Figure 4.** Calcium ion chelating capacity analysis based on x-ray photoelectron spectroscopy spectra. a) Full x-ray photoelectron spectroscopy spectra of DCLS and HCLS treated with 0.3M $CaCl_2$ solution. b) c) High-resolution x-ray photoelectron spectroscopy spectra of C1s and O1s in DCLS-0.3M$Ca^{2+}$ and HCLS-0.3M$Ca^{2+}$.

The freeze-dried HCLS was immersed in 1.5×SBF at 37° C for biomimetic apatite deposition. The SBF was updated every day to maintain a consistent ionic strength. After 14 days, this scaffold was removed from the SBF, washed gently with DDW, and then freeze-dried.

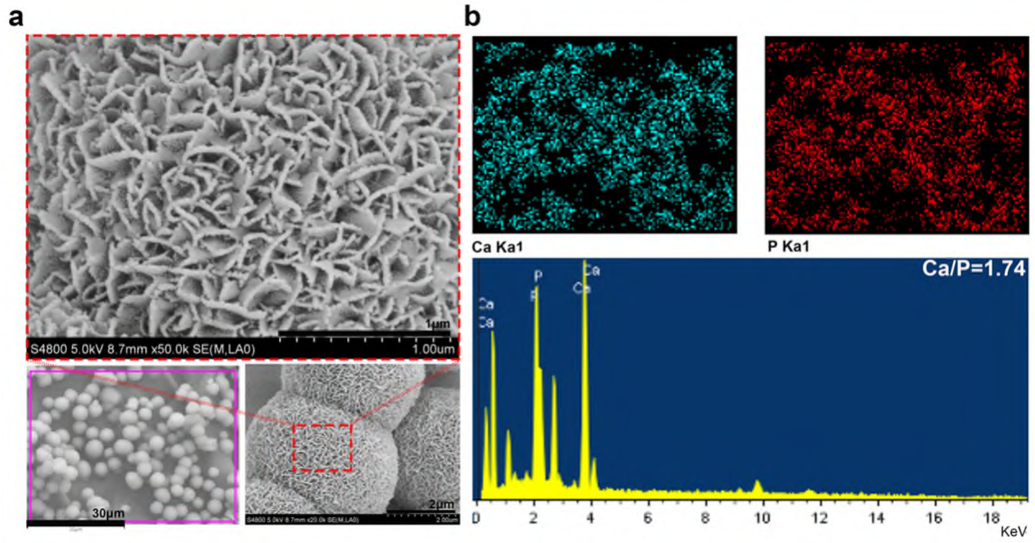


**Supplementary Figure 5.** Calcium chelation and mineralization of HCLS in SBF at 37℃ for 7 days. a) SEM image of bone-like apatite at different magnifications in HCLS. b) EDS evaluation of Ca/P ratios and element.

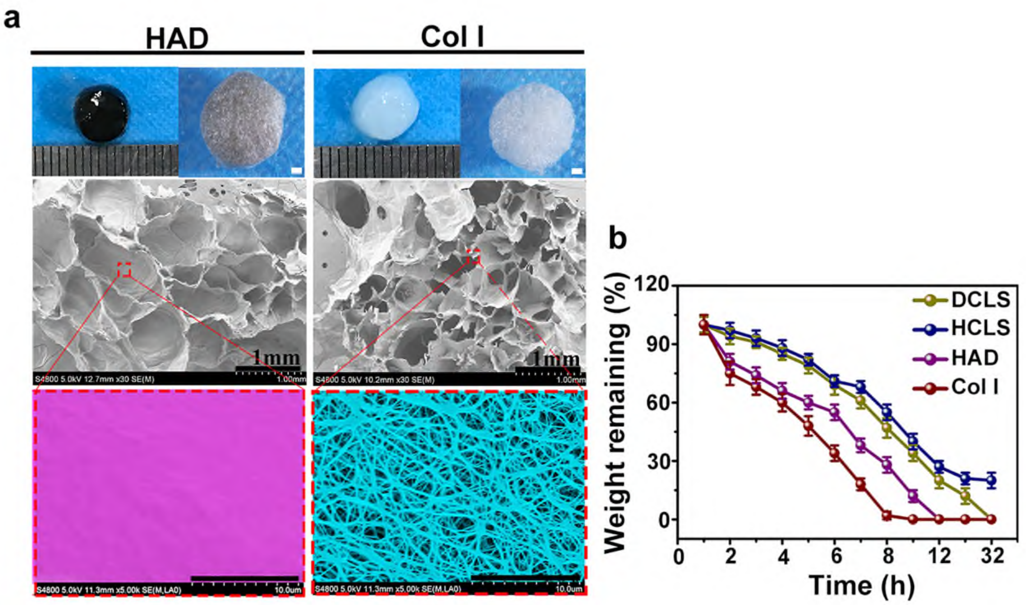


**Supplementary Figure 6.** Characterization of scaffolds. a) Gross appearance of various hydrogels and their freeze-dried scaffolds and SEM images of their inner sections. b) Enzymatic degradation of hydrogels against hyaluronidase (100 U/mL) and type I collagenase (100 U/mL). (Error bars represent standard deviation, n = 3 independent replicates).

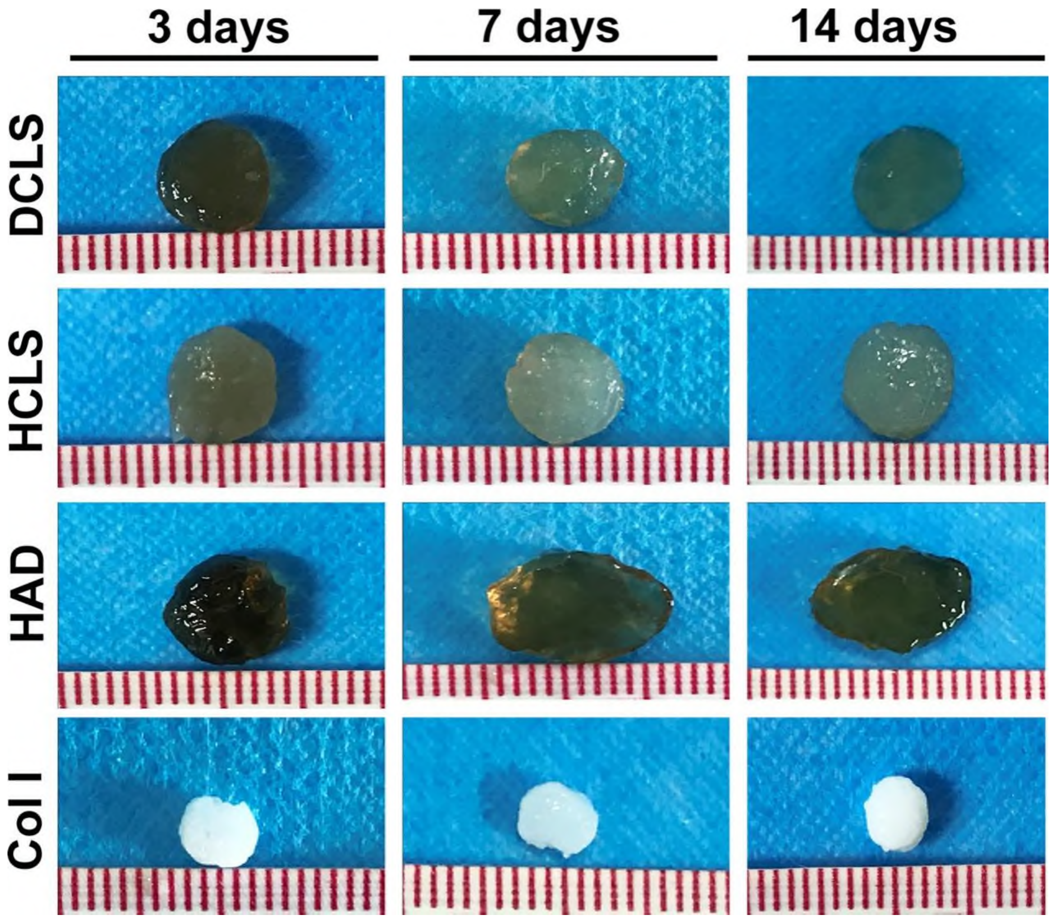


**Supplementary Figure 7.** *In vitro* swelling test in Dulbecco's phosphate-buffered saline (DPBS) for DCLS, HCLS, HAD and Col I at 3, 7 and 14days.

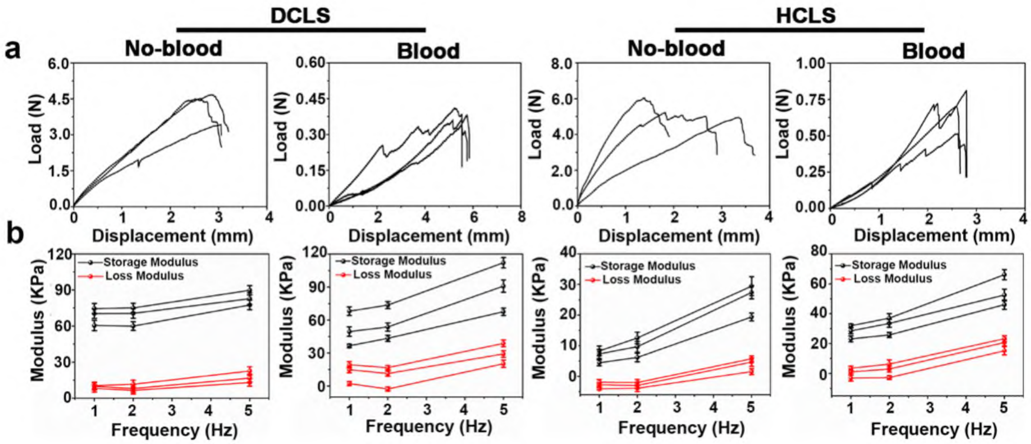


**Supplementary Figure 8.** The original data of mechanical characterization of self-adhesive and flexible scaffold. a) Tensile test of various scaffolds with or without invasive blood. b) Characterization of compressive storage modulus with or without

invasive blood. (Error bars represent standard deviation, n = 3 independent replicates).

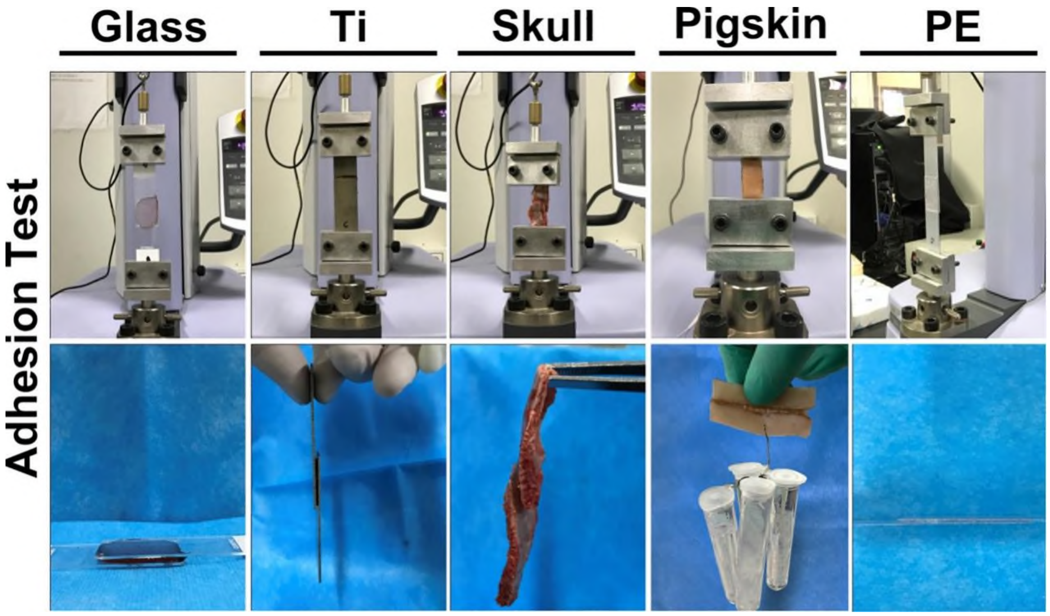


**Supplementary Figure 9.** Adhesion test of DCLS, HCLS, HAD and Col I on different substrates including glass, Ti, skull, pigskin and PE.

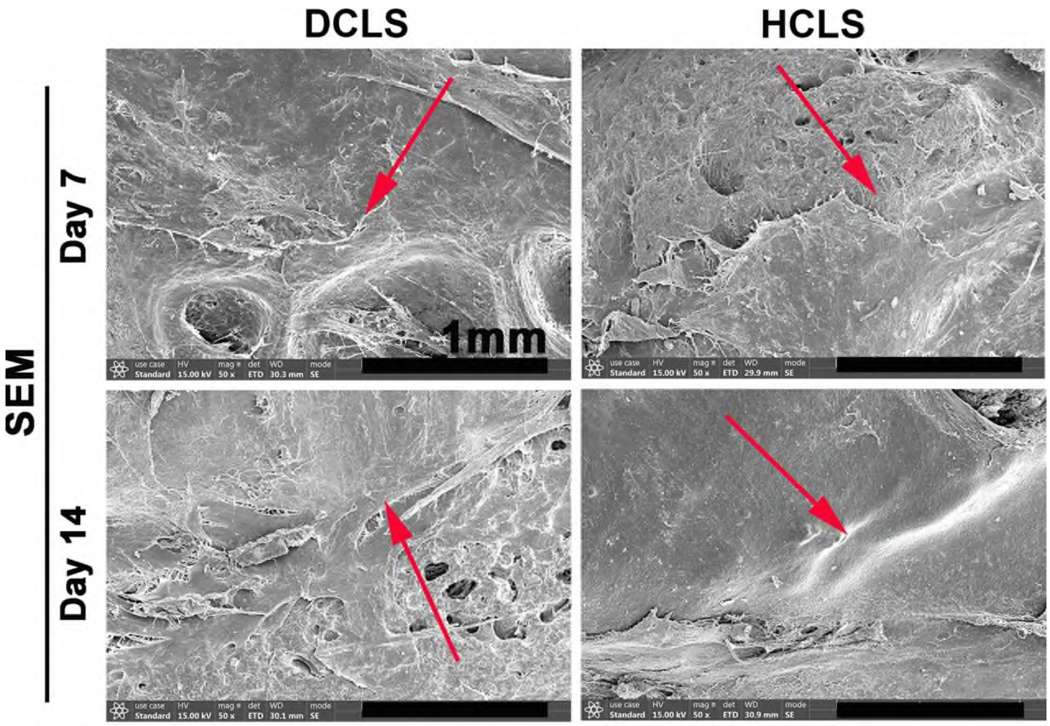


**Supplementary Figure 10.** SEM images of the interface between scaffold and host

bone tissue on day 7 and 14.

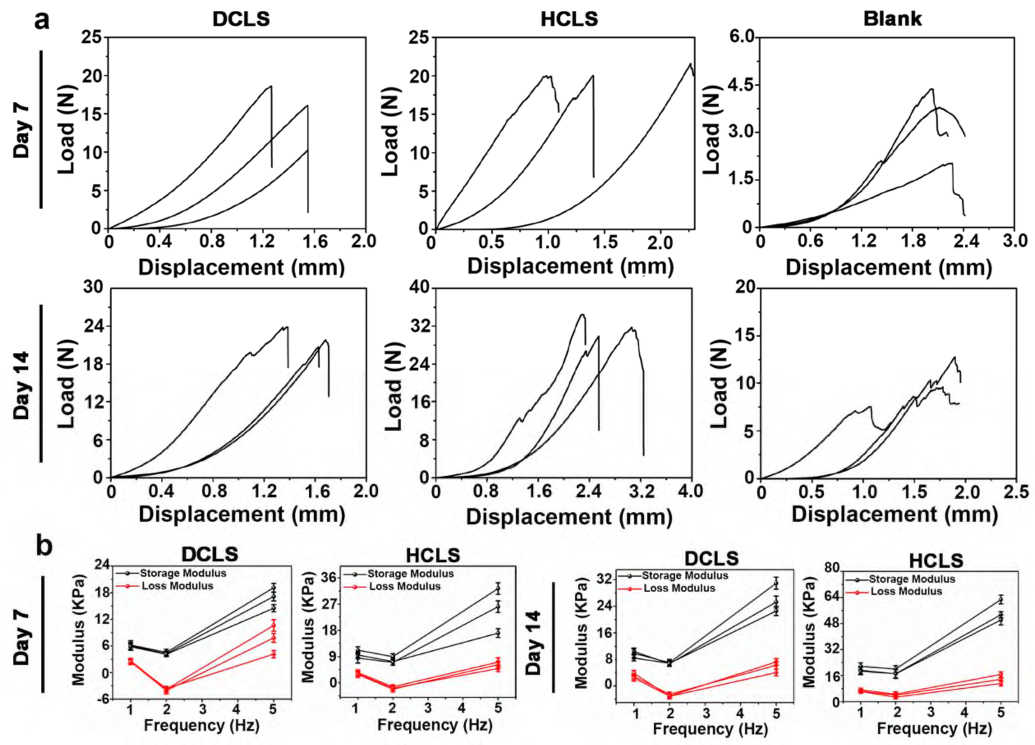


**Supplementary Figure 11.** The original data of *in vivo* mechanical characterization of scaffold after implantation in rabbit skull for 7 and 14 days. a) Tensile test of various implants on day 7 and 14. b) Characterization of compressive storage modulus of various implants on day 7 and 14. (Error bars represent standard deviation, n = 3 independent replicates).

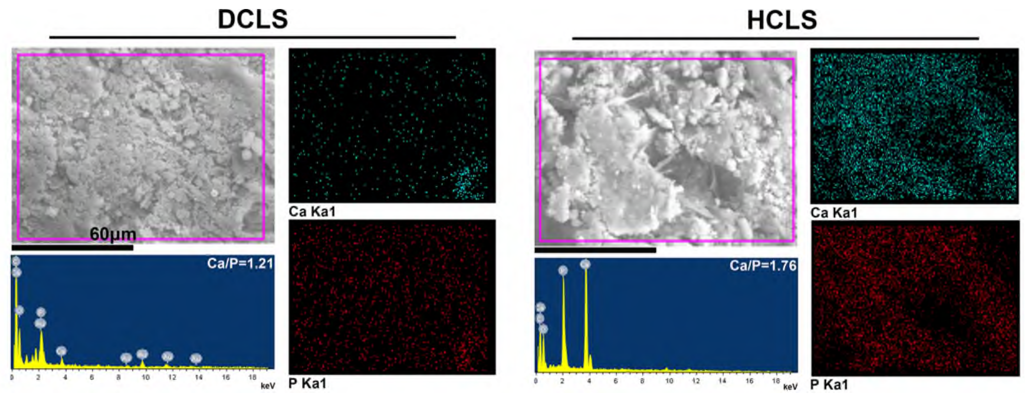


**Supplementary Figure 12.** Ca/P ratios and element analysis at the interface between implant and host bone based on EDS analysis after implanting in rabbit cranial defect.

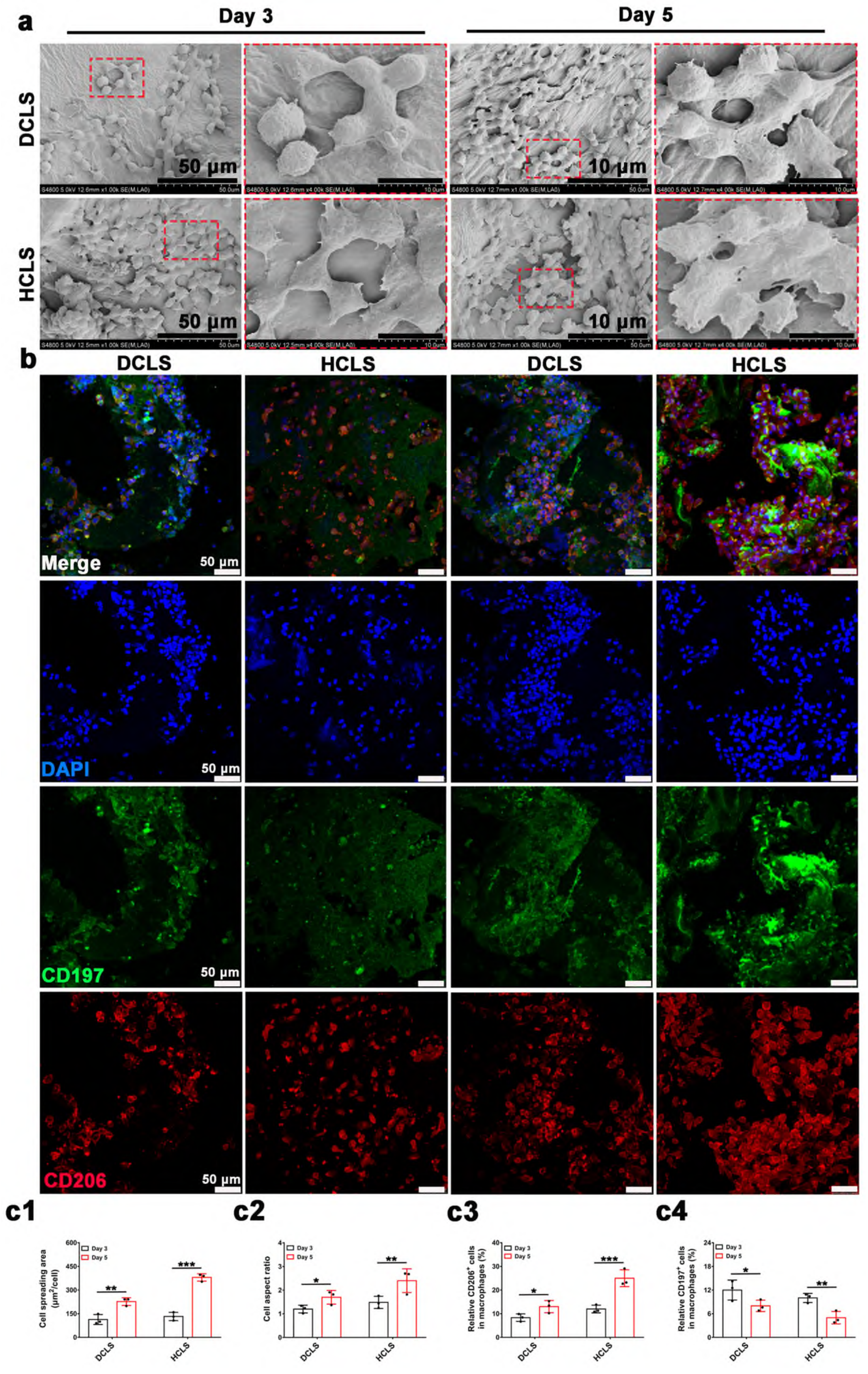


**Supplementary Figure 13.** Response of scaffolds to macrophages *in vitro*. a) Cell

morphologies of macrophages on different samples on day 3 and 5. b) CLSM images of CD206, CD97 and nucleic staining of macrophages on different samples on day 3 and 7. c1) Quantitative cell spreading area based on SEM images (**p=0.0073, ***p=7.4781×10$^{-5}$). c2) Quantitative cell aspect ratio based on SEM images (*p=0.0422, **p=0.0054). c3) c4) Relevant semi-quantitative analysis of immunofluorescence staining by Image J software (c3: *p=0.0322, ***p=0.0003; c4: *p=0.0418, **p=0.0065). n = 3 cells examined 3 independent experiments. (Two-sided comparison, Error bars represent standard deviation, *p < 0.05, **p < 0.01, and ***p < 0.001).

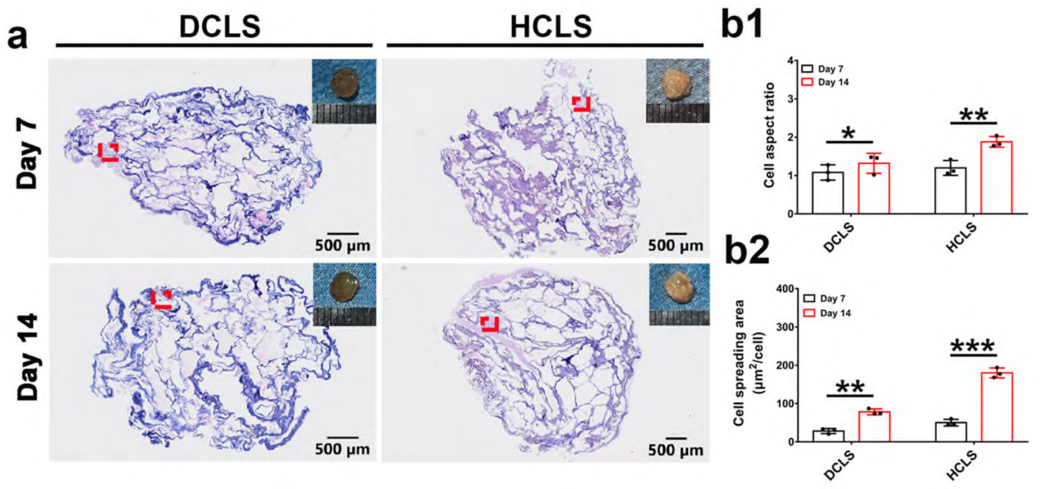


**Supplementary Figure 14.** *In vivo* immune response of scaffold in mouse intramuscular and rabbit skull defect model. a) Hematoxylin and eosin (H&E) staining of implants on days 7 and 14 after implantation in mouse intramuscular. Insets are macroscopic views of the indicated implants. b1) Quantitative cell aspect ratio based on SEM images (*p=0.0487, **p=0.0077). b2) Quantitative cell spreading area based on SEM images (**p=0.0069, ***p=0.0005). n = 3 cells examined 3 independent experiments. (Two-sided comparison, Error bars represent standard deviation, *p < 0.05, **p < 0.01, and ***p < 0.001).

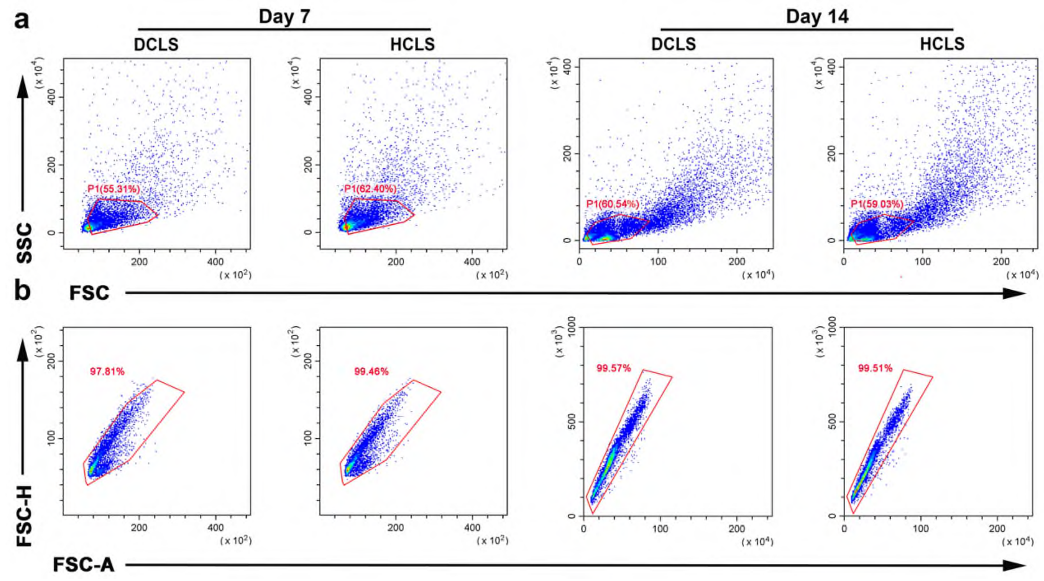


**Supplementary Figure 15.** Flow cytometry data analysis. a) The initial gating involved exclusion of debris with FSC/SSC by excluding low FSC/SSC values (extreme lower left quadrant). b) Positive selection for live cells, with a subsequent gate applied to select for macrophage cells while excluding other cells.

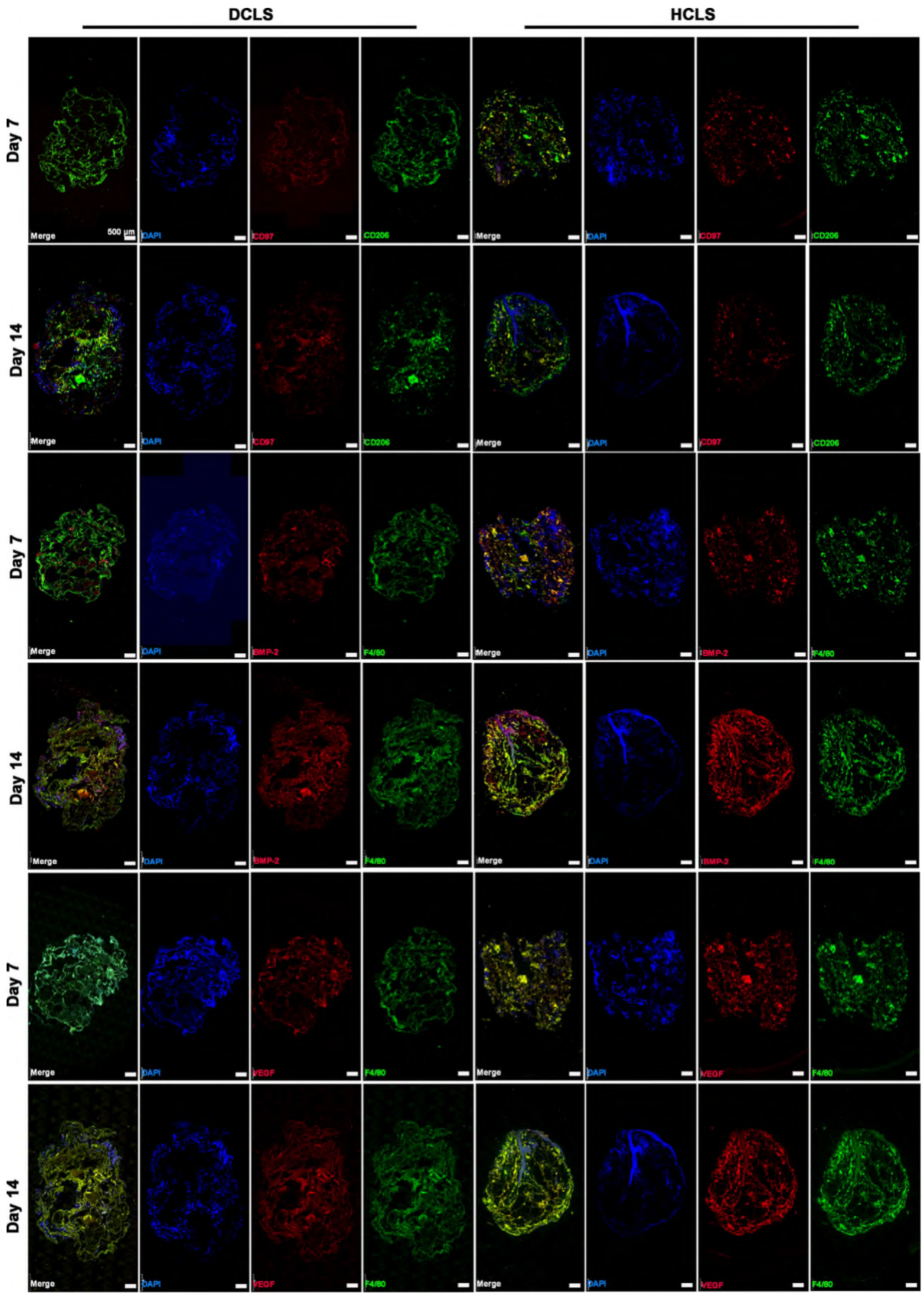


**Supplementary Figure 16.** Representative immunostainings of CD197 and CD206, BMP-2 secretion and F4/80$^{+}$, and VEGF secretion and F4/80$^{+}$ macrophages in DCLS and HCLS on days 7 and 14 after implantation in mouse intramuscular.

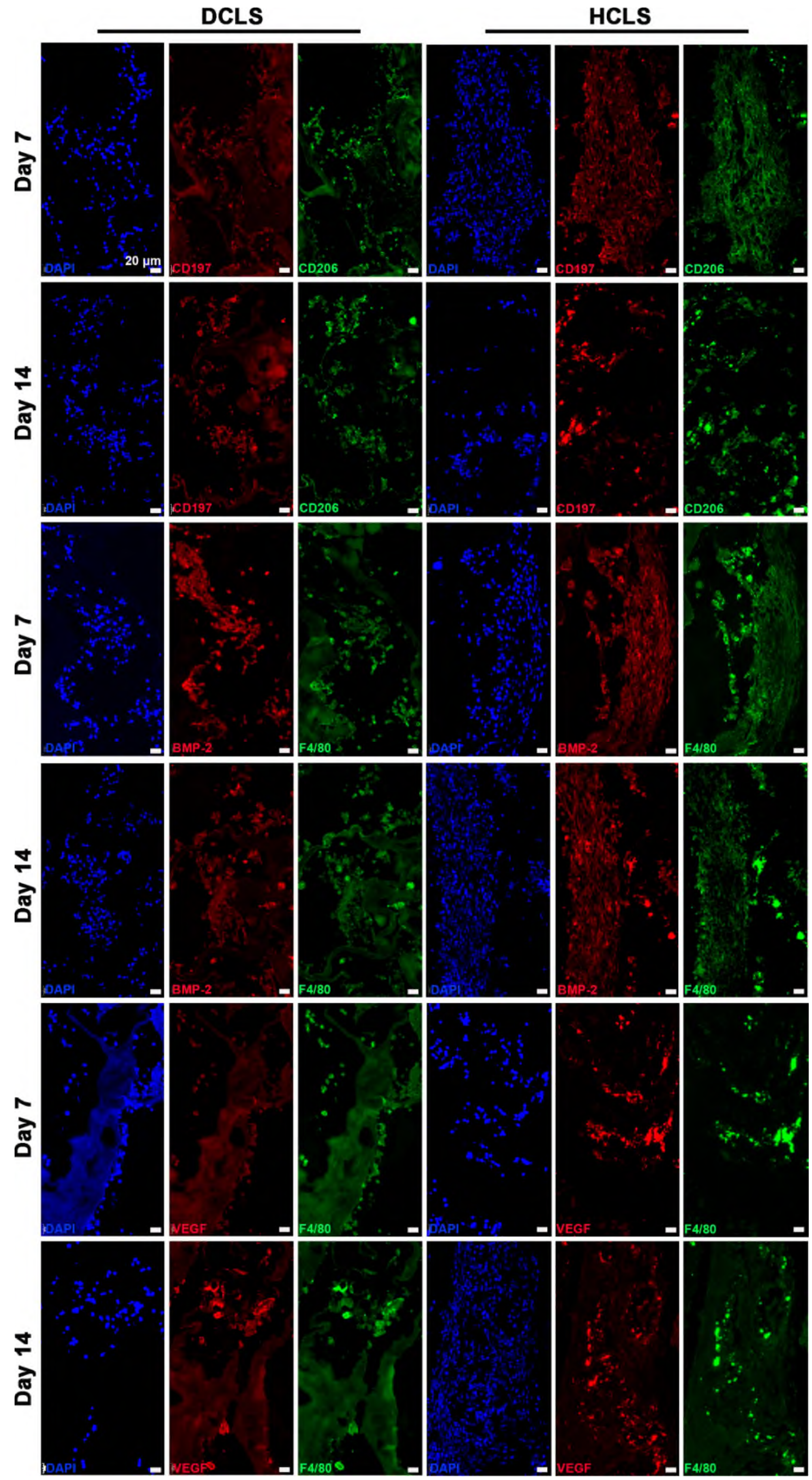


**Supplementary Figure 17.** Representative local amplification immunostainings of CD197 and CD206, BMP-2 secretion and F4/80$^+$, as well as VEGF secretion and F4/80$^+$ macrophages in DCLS and HCLS on day 7 and 14 after intramuscular implantation in mouse.

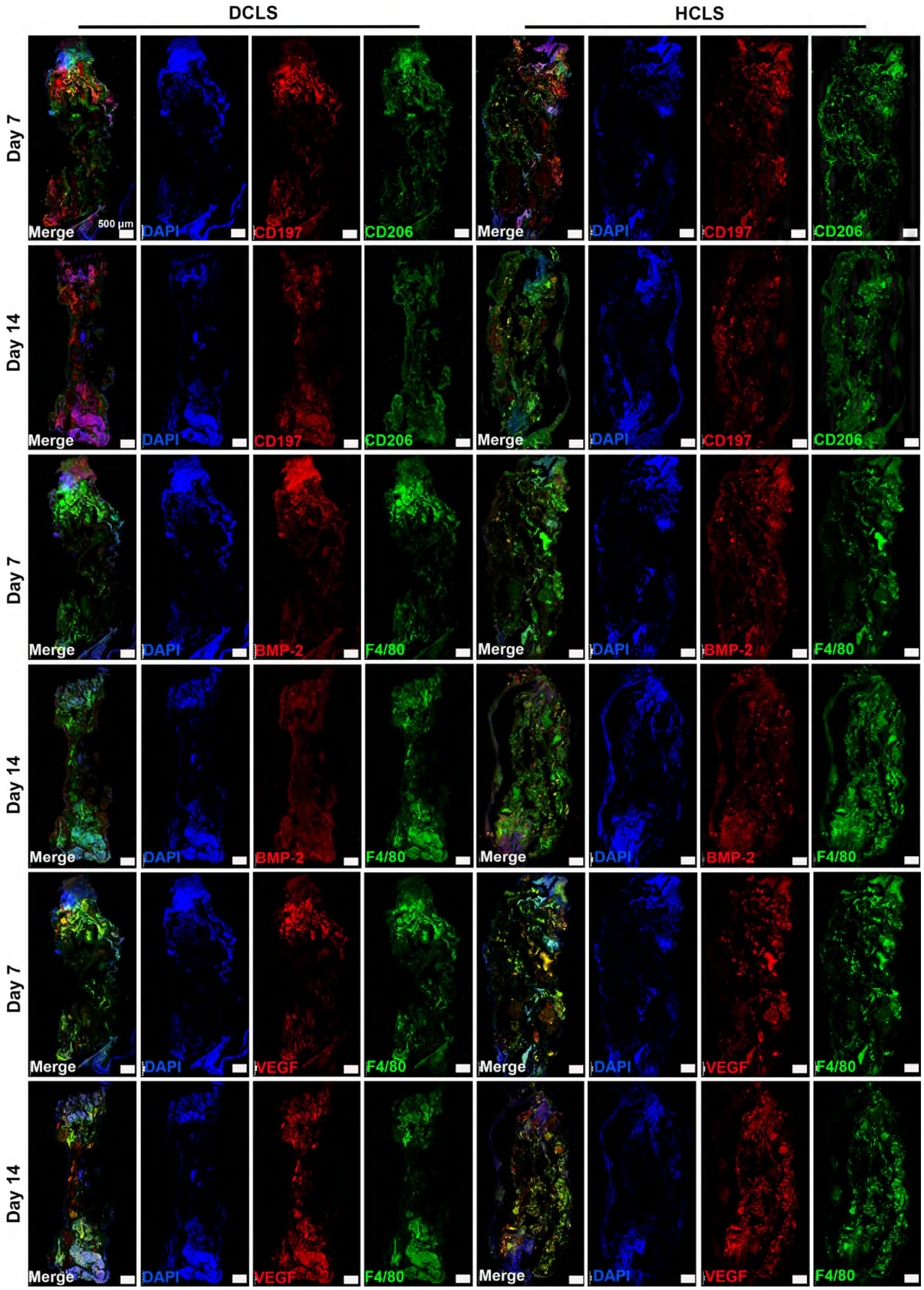


**Supplementary Figure 18.** Representative immunostainings of CD197 and CD206, BMP-2 secretion and F4/80$^+$, and VEGF secretion and F4/80$^+$ macrophages in DCLS and HCLS on days 7 and 14 after implantation in rabbit skull defect model.

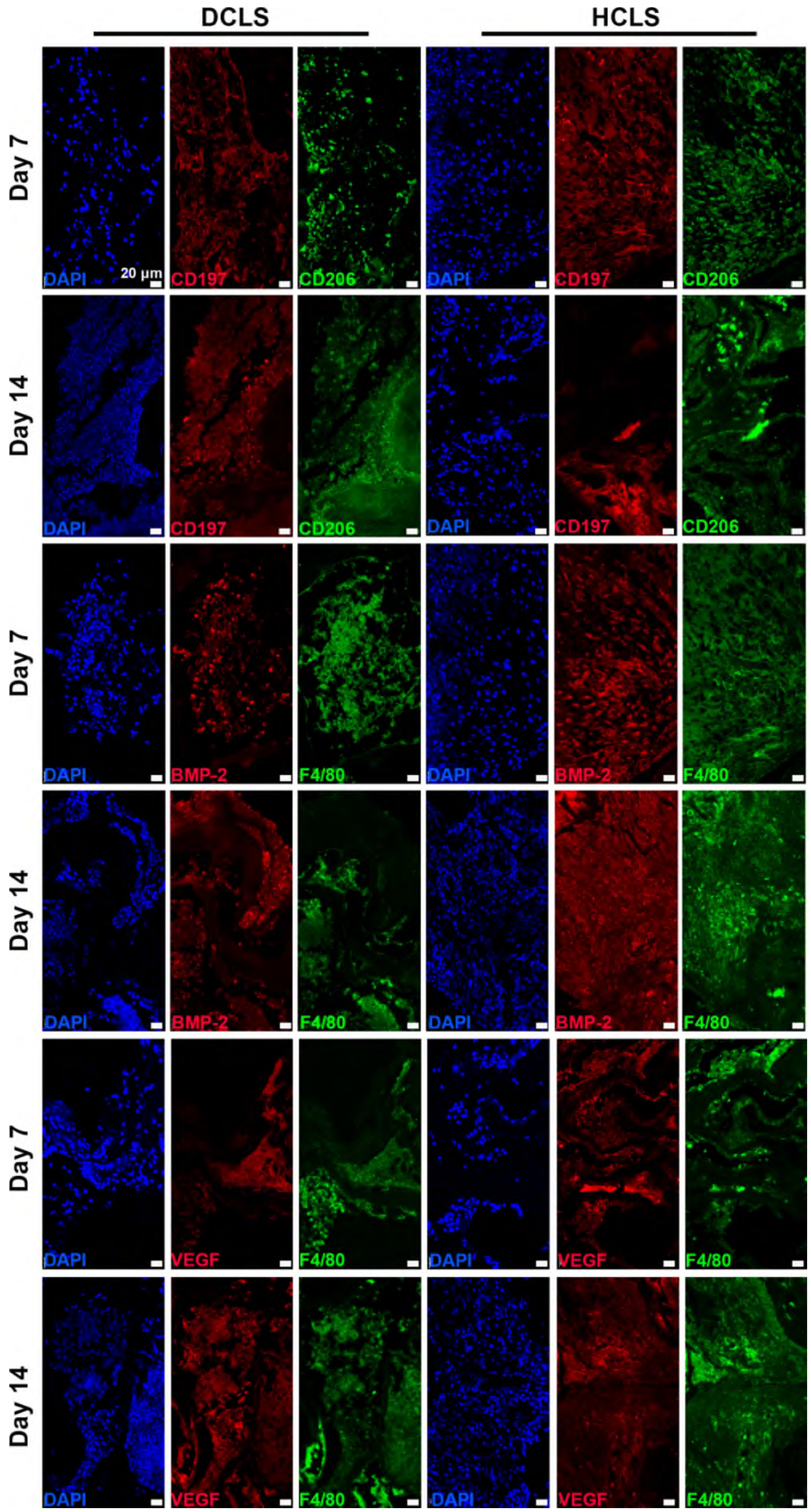


**Supplementary Figure 19.** Representative local amplification immunostainings of CD197 and CD206, BMP-2 secretion and F4/80$^+$, and VEGF secretion and F4/80$^+$ macrophages in DCLS and HCLS on days 7 and 14 after implantation in rabbit skull defect model.

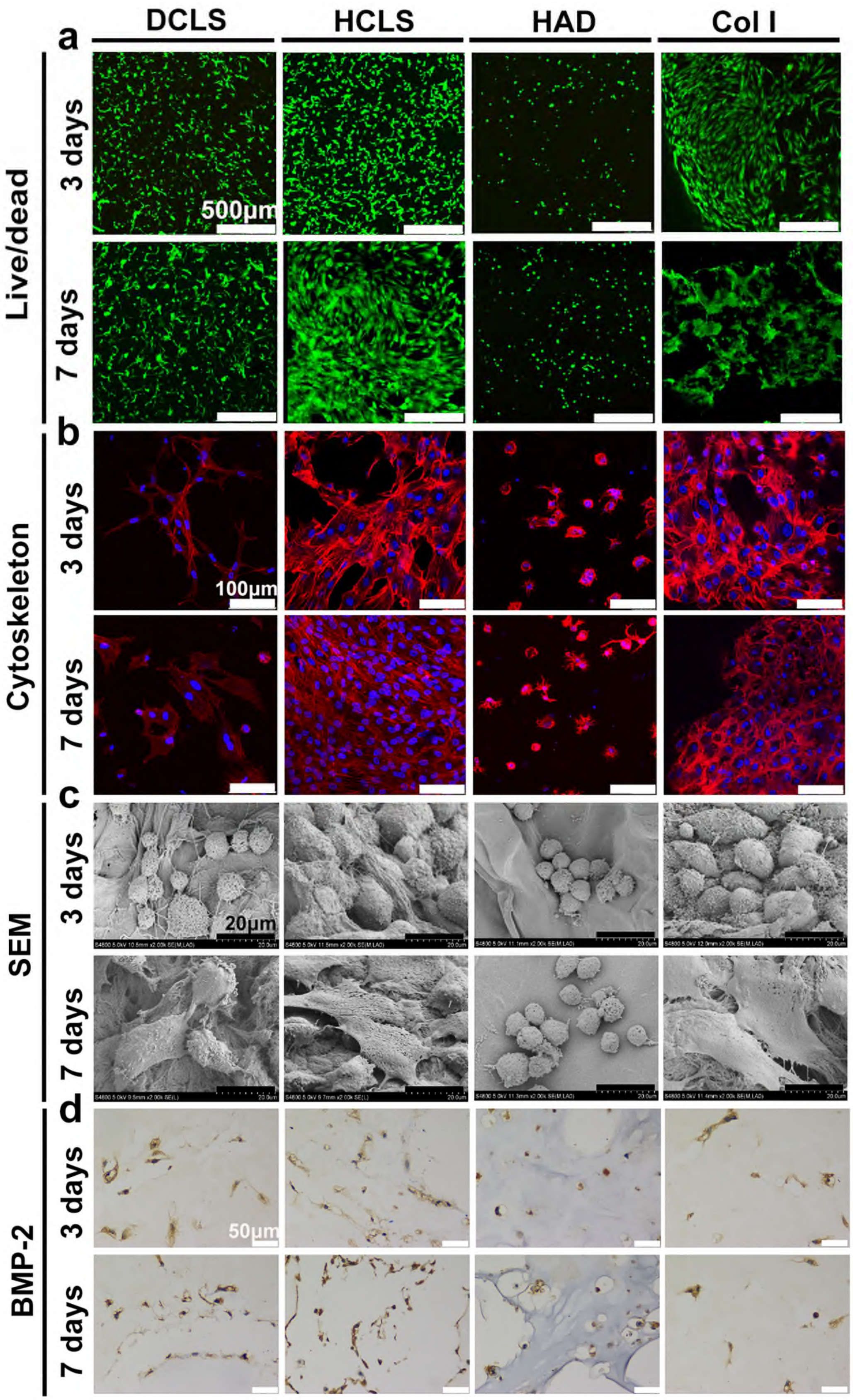
DCLS
HCLS
HAD
Col I
a
b
c
d
Live/dead
Cytoskeleton
SEM
BMP-2
3 days
7 days
500μm
100μm
20μm
50μm

**Supplementary Figure 20.** Proliferation and morphology of BMSCs within DCLS, HCLS, HAD and Col I. a) CLSM images (FDA/PI staining) of BMSCs encapsulated into various hydrogels at day 3 and 7. b) CLSM images (rhodamine-phalloidin/DAPI staining) of BMSCs at day 3 and 7.c) SEM images of BMSCs at day 3 and 7. d) Immunohistochemistry staining of BMP-2 at day 3 and 7.

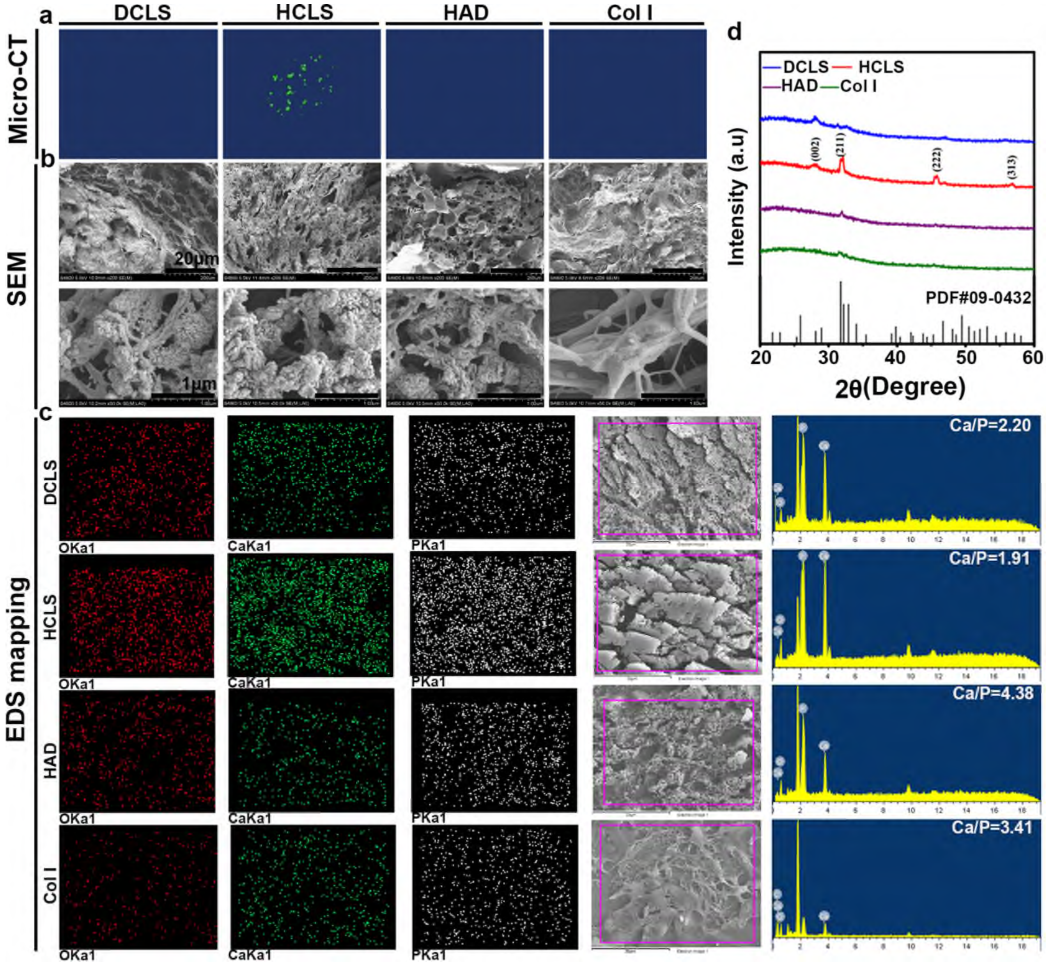


**Supplementary Figure 21.** Characterization of various specimens after 30 days subcutaneous implantation in nude mice. a) Micro-CT 3D reconstruction images of DCLS, HCLS, HAD and Col I before implantation. b) SEM images of inner section of BMSCs-laden DCLS, HCLS, HAD and Col I after 30 days subcutaneous implantation in nude mice. c) EDS analysis of Ca/P ratio and distribution of various samples after 30 days subcutaneous implantation in nude mice. d) XRD analysis of mineralized bone tissue in various specimens after 30 days subcutaneous implantation in nude mice.

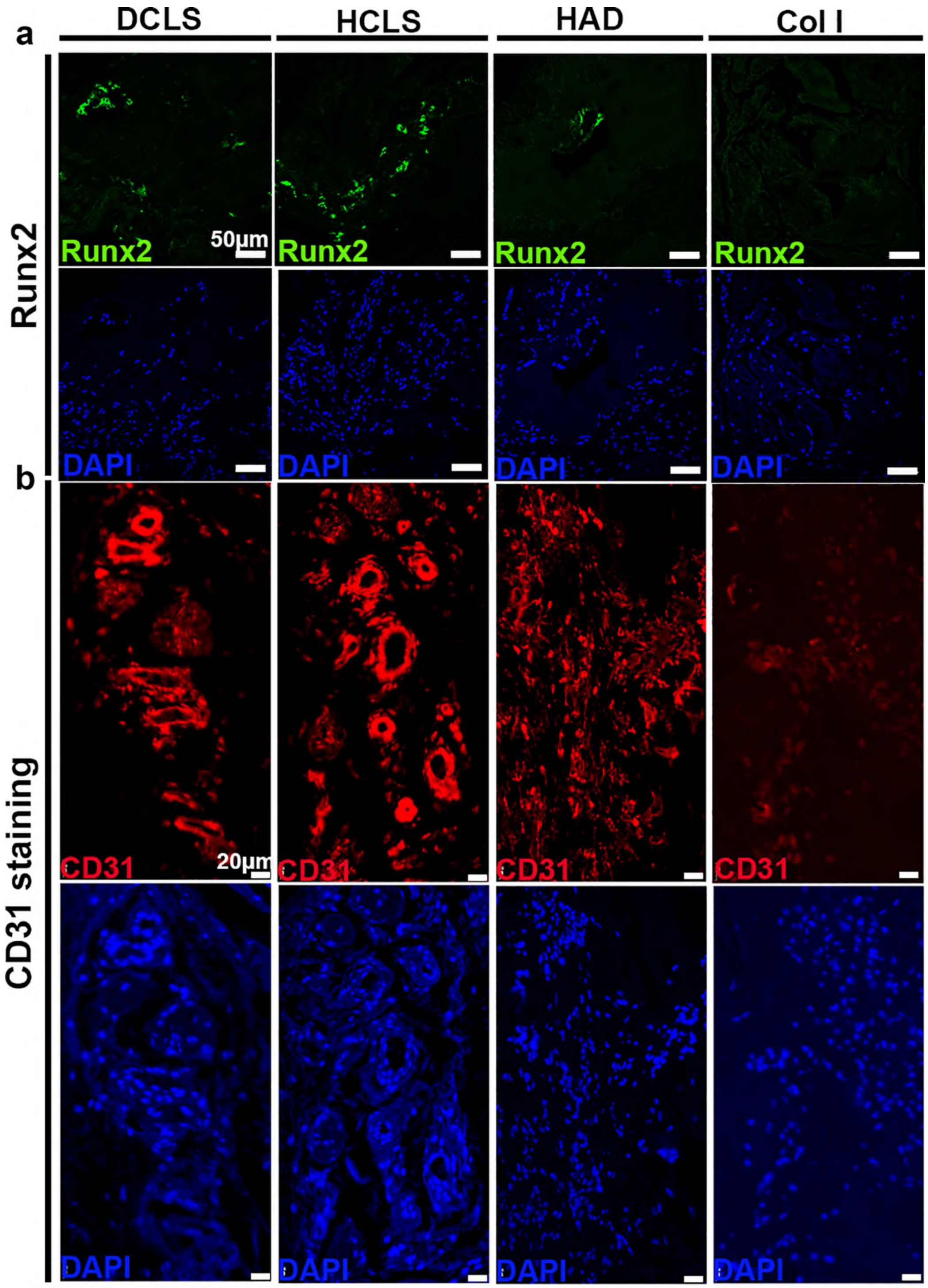


**Supplementary Figure 22.** Immunofluorescence staining of Runx2 and CD31 after 30 days subcutaneous implantation in nude mice. a) Immunofluorescence staining of Runx2 of various samples after 30 days subcutaneous implantation in nude mice. b) Immunofluorescence staining of CD31 of various samples after 30 days subcutaneous implantation in nude mice.

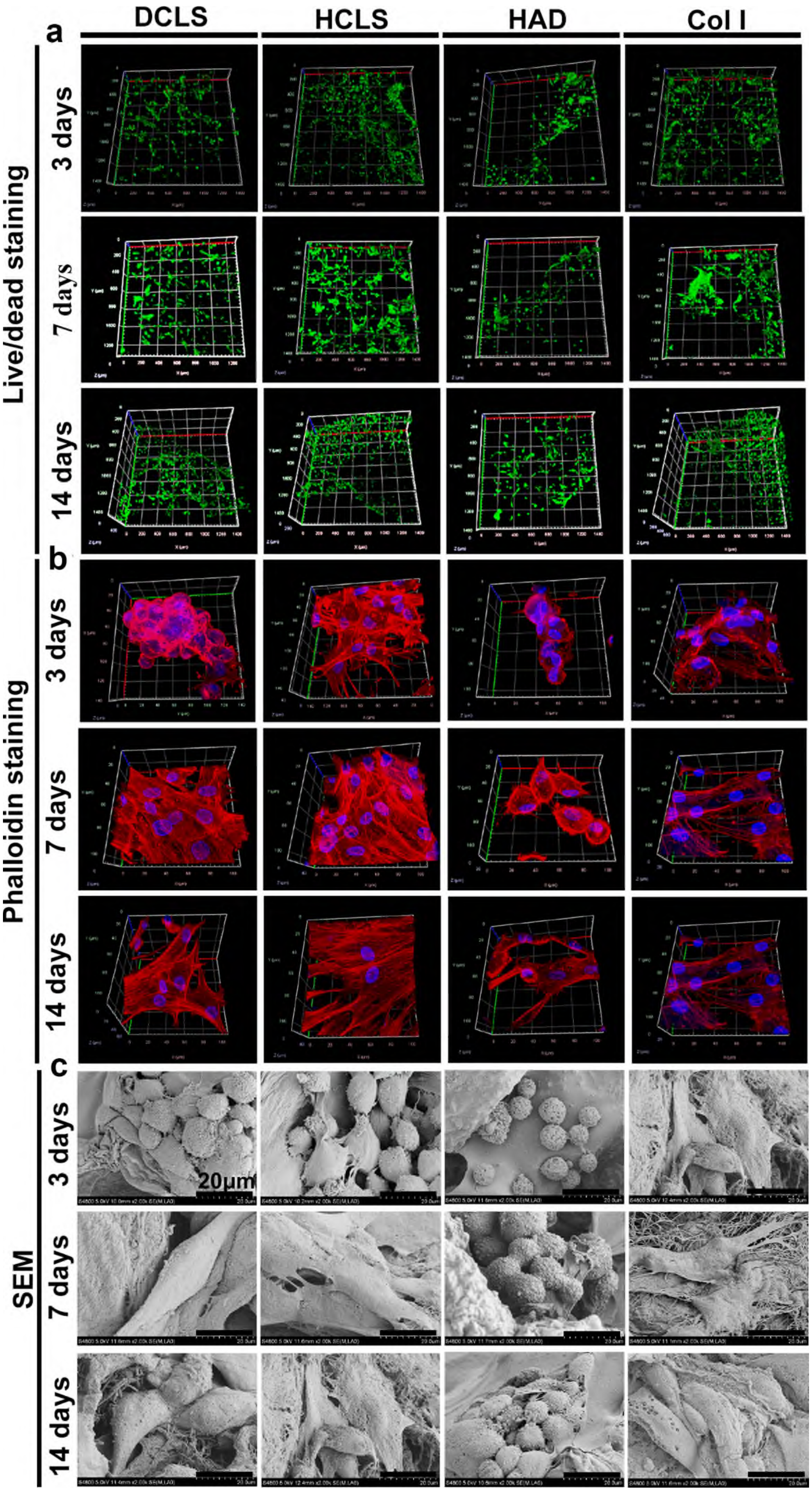
DCLS
HCLS
HAD
Col I
a
b
c
Live/dead staining
Phalloidin staining
SEM
3 days
7 days
14 days
20μm

**Supplementary Figure 23.** *In vitro* BMSCs retention by DCLS, HCLS, HAD and Col I. a) CLSM images (FDA/PI staining) of BMSCs seeded onto the surface of various scaffolds at day 3 and 7. b) CLSM images (rhodamine-phalloidin/DAPI staining) of BMSCs seeded onto the surface of various scaffolds at day 3 and 7. c) SEM images of BMSCs seeded onto the surface of various scaffolds at day 3 and 7.

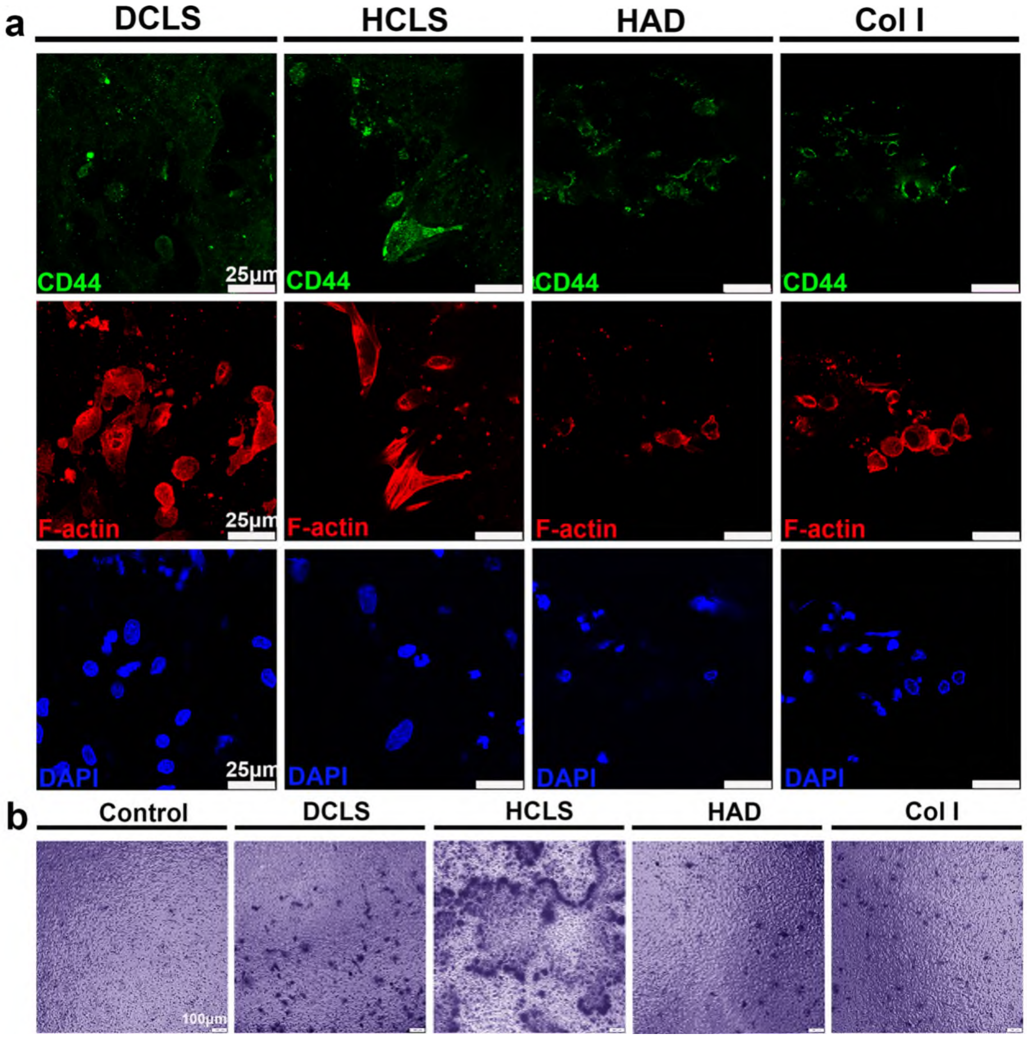


**Supplementary Figure 24.** *In Vitro* BMSCs recruitment by DCLS, HCLS, HAD and Col I. a) CD44 immunofluorescent staining of various samples imaged by CLSM after incubating in a whole rabbit cranial bone marrow cell suspension for 48h. b) Transwell-migration assay. BMSCs were seeded on the upper chamber and the DCLS, HCLS, HAD and Col I scaffolds were placed in the lower chambers. After 12 h incubation, cells that traverse the membrane were stained with 0.1% crystal violet and imaged by a microscope (scale bars: 100 μm);

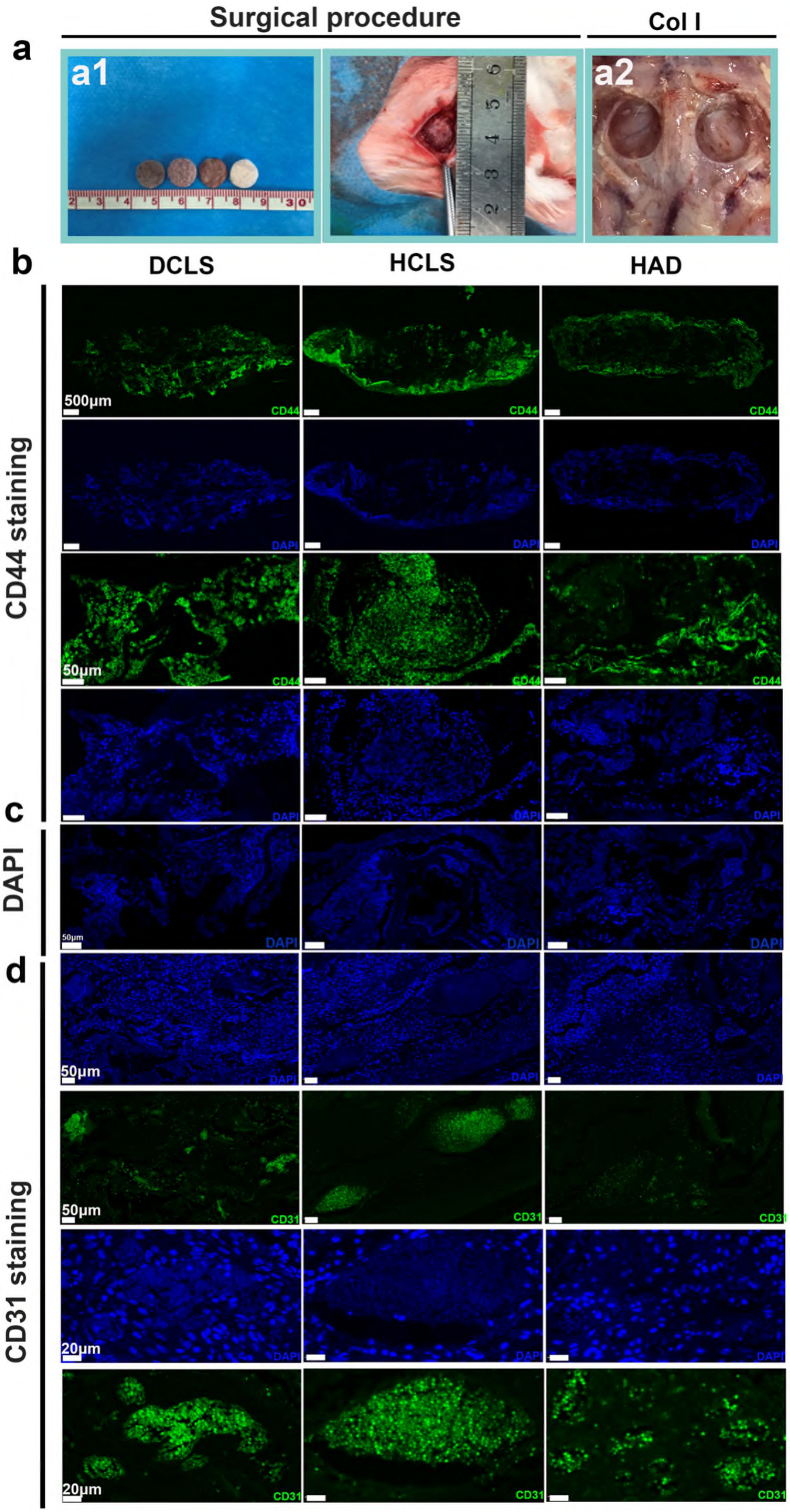

a
Surgical procedure
Col I
a1
a2
b
DCLS
HCLS
HAD
CD44 staining
500μm
50μm
CD44
DAPI
c
DAPI
50μm
d
CD31 staining
50μm
20μm
CD31

**Supplementary Figure 25.** *In vivo* cell recruitment by DCLS, HCLS, HAD and Col I in a rabbit cranial defect model (Φ = 9 mm) after one week's implantation. a1) Surgical procedure. a2) Expalnts of Col I group after one week's implantation. b) Dual turntable confocal scanning images (CD44/DAPI staining) of ECSs in various samples. c) DAPI staining of BMP-2 and Runx2 immunofluorescence staining at one week of different treatments. d) Confocal Laser scanning microscope images of CD31 immunofluorescence staining of various samples after one week's implantation.

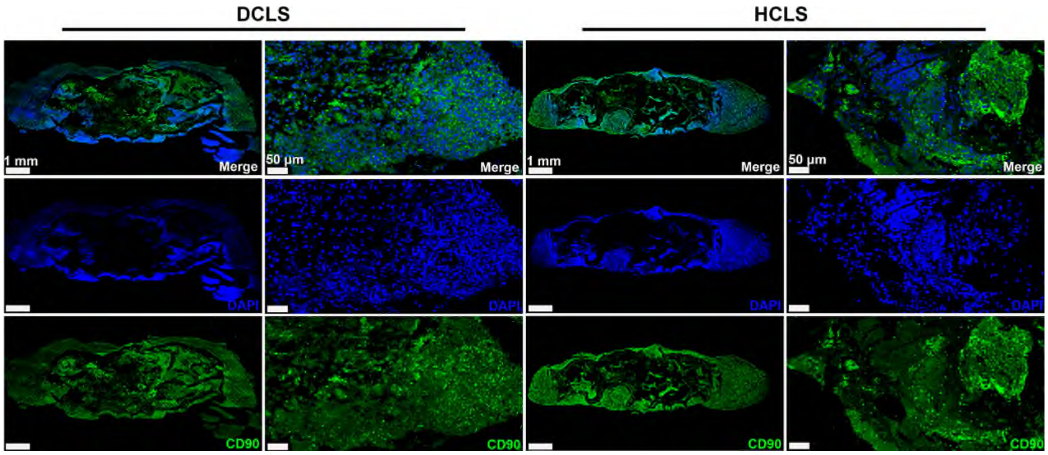


**Supplementary Figure 26.** CD90 immunofluorescence staining of BMSCs in scaffolds at one week after implantation in the rabbit cranial defect.

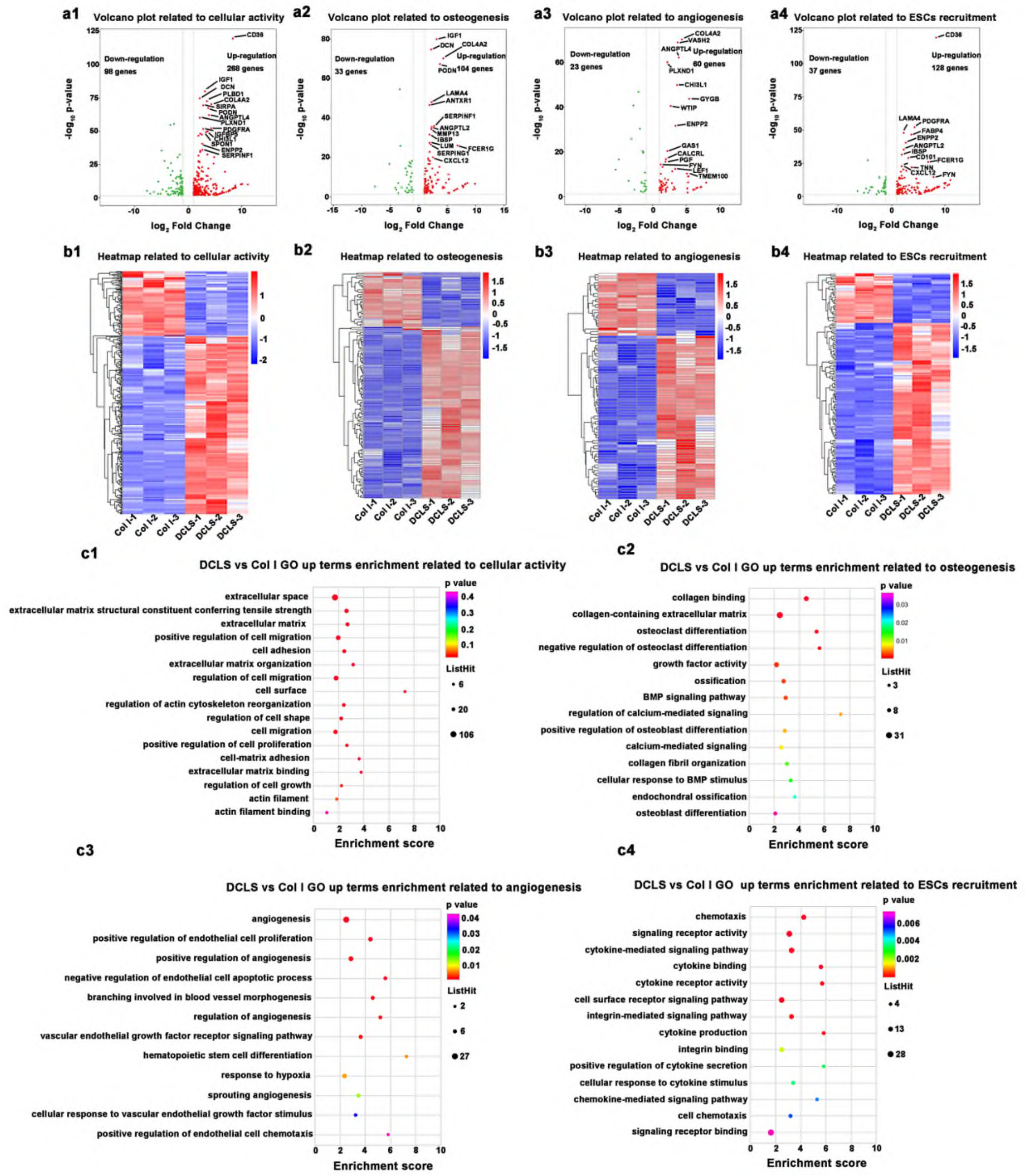


**Supplementary Figure 27.** Self-adhesive and flexible scaffold regulates gene expressions related to cellular activity, osteogenesis, angiogenesis, and ESCs recruitment on DCLS versus Col I. a1-4) Volcano plot of transcriptomic analysis of differentially expressed genes related to cellular activity, osteogenesis, angiogenesis, and ESCs recruitment. b1-4) Heatmap analysis of differentially expressed genes involved in cellular activity, osteogenesis, angiogenesis, and ESCs recruitment. c1-4) Enriched GO up terms of DCLS versus Col I related to cellular activity, osteogenesis, angiogenesis, and ESCs recruitment. n = 3 independent experiments per group.

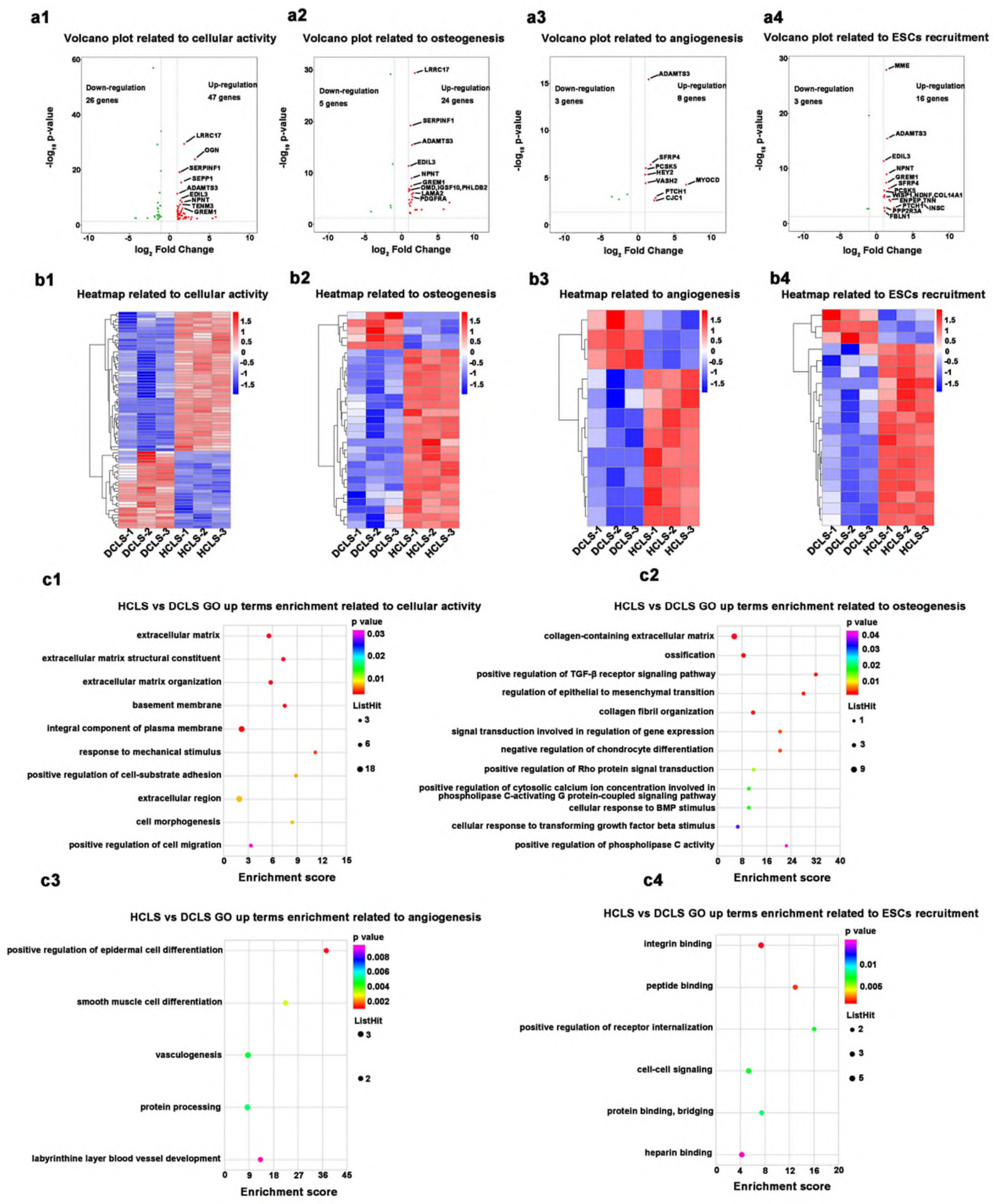


**Supplementary Figure 28.** Self-adhesive and flexible scaffold regulates gene expressions related to cellular activity, osteogenesis, angiogenesis, and ESCs recruitment on HCLS versus DCLS. a1-4) Volcano plot of transcriptomic analysis of differentially expressed genes related to cellular activity, osteogenesis, angiogenesis, and ESCs recruitment. b1-4) Heatmap analysis of differentially expressed genes involved in cellular activity, osteogenesis, angiogenesis, and ESCs recruitment. c1-4) Enriched GO up terms of HCLS versus DCLS related to cellular activity, osteogenesis, angiogenesis, and ESCs recruitment. n = 3 independent experiments per group.

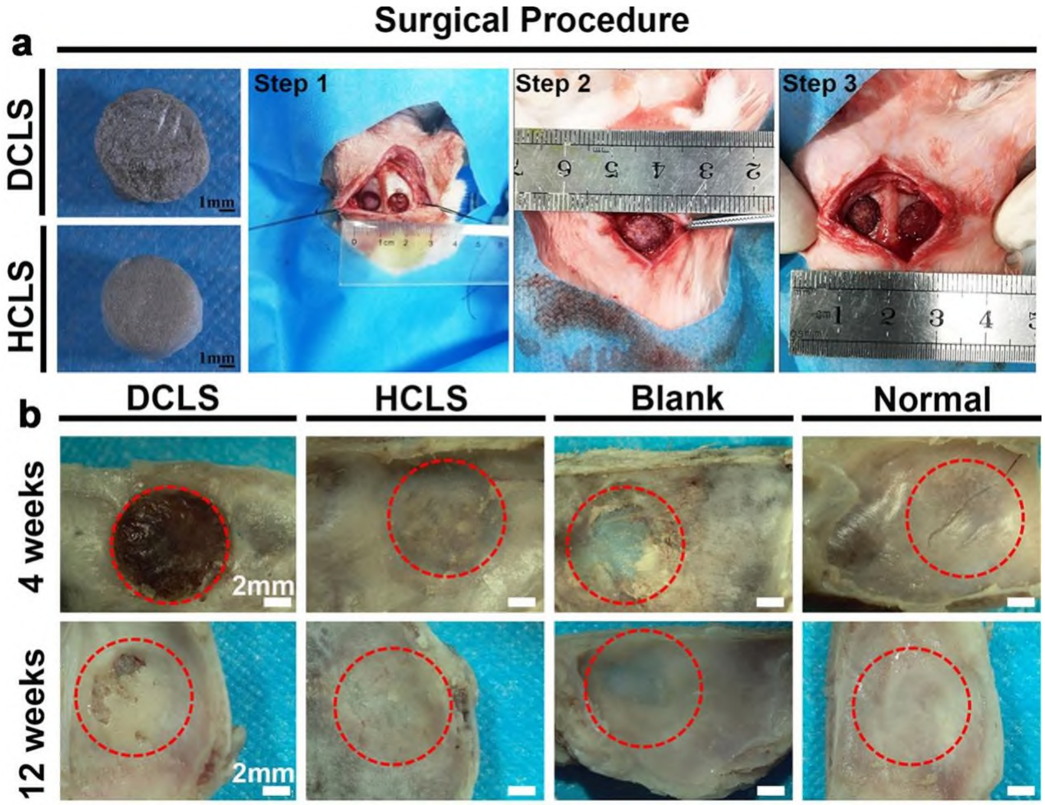


**Supplementary Figure 29.** In situ visual skull reconstruction effects of DCLS and HCLS in rabbit cranial defect model (Φ = 9 mm). a) Surgical procedures. b) Gross appearance of the regenerated tissue at 4 and 12 weeks of different treatments.

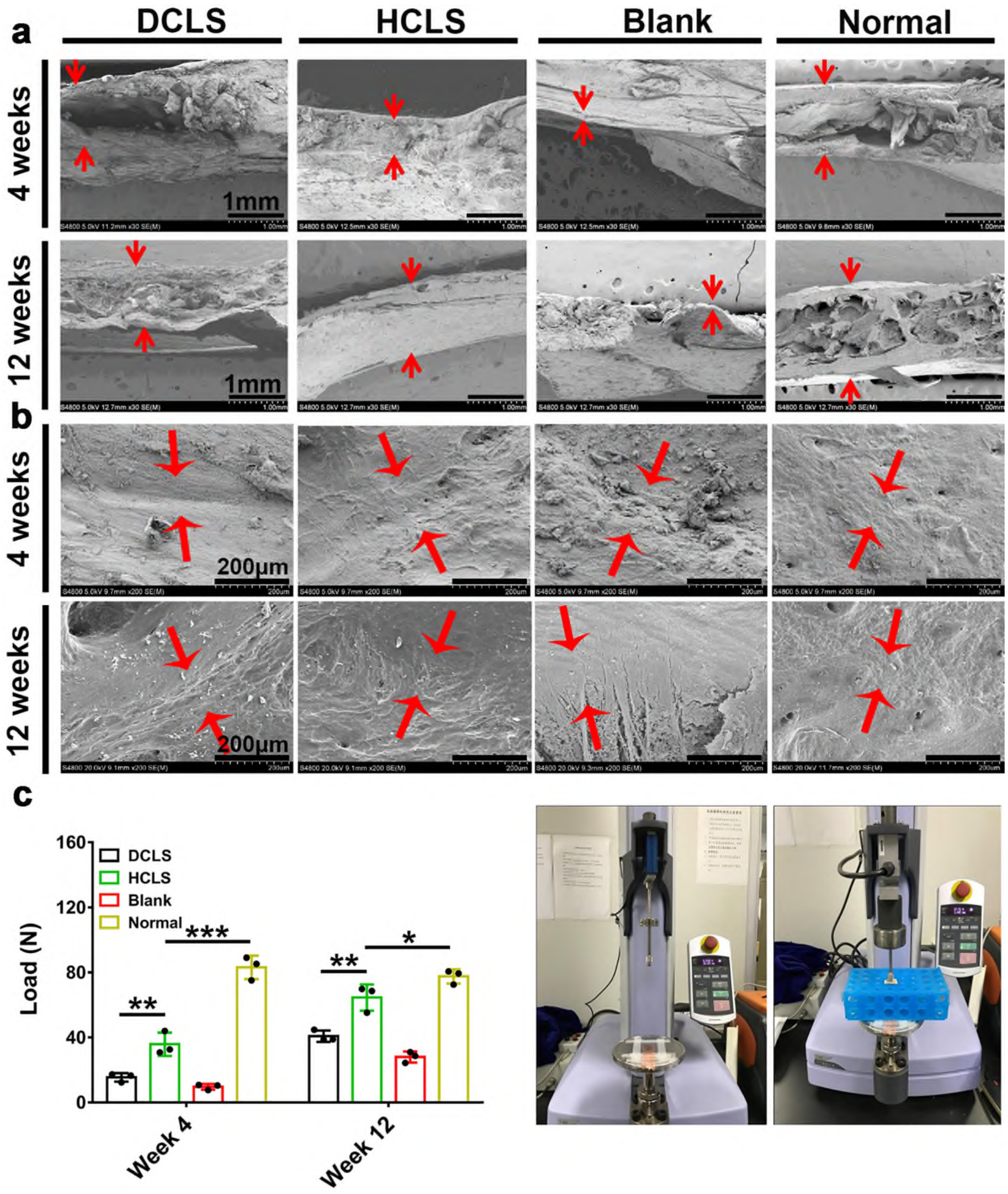


**Supplementary Figure 30.** Characterization of various explants at week 4 and 12 after implantation in rabbit cranial defect model (Φ = 9 mm). a) SEM images of cross section of the defect of various treatment groups at week 4 and 12. b) SEM images of the interface between host tissue and various scaffolds in the defect at week 4 and 12. c) Push-out test of various treatment groups at week 4 and 12 (**p=0.0019, ***p=$6.8344\times10^{-6}$, **p=0.0045, *p=0.0388). n = 3 biologically independent samples. (Two-sided comparison, Error bars represent standard deviation, *$p < 0.05$, **$p < 0.01$, and ***$p < 0.001$).

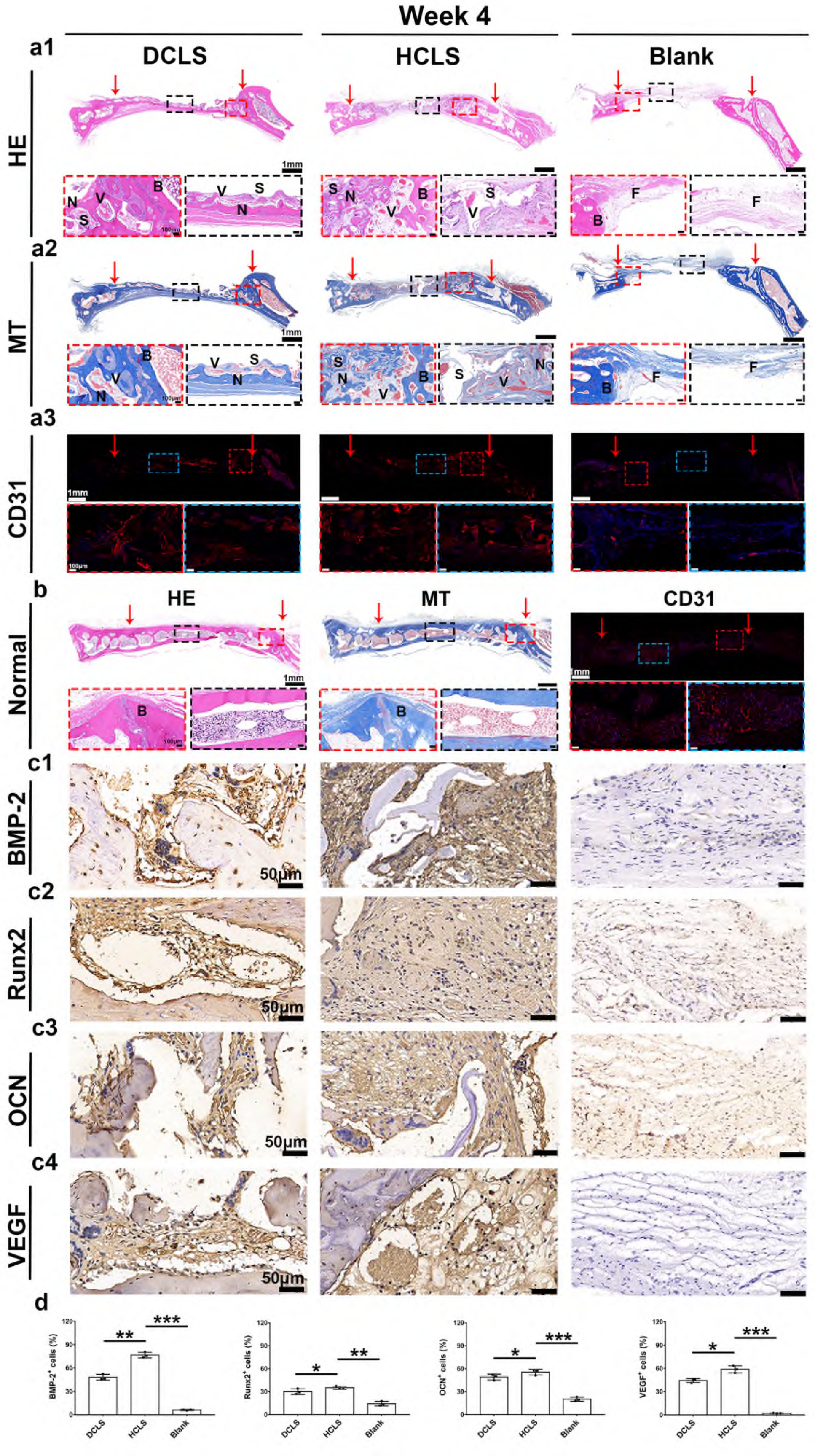
Week 4
a1
DCLS
HCLS
Blank
HE
a2
MT
a3
CD31
b
HE
MT
CD31
Normal
c1
BMP-2
c2
Runx2
c3
OCN
c4
VEGF
50μm
d
DCLS
HCLS
Blank

**Supplementary Figure 31.** Representative staining images of regenerated tissue at week 4. a1) H&E staining of regenerated bones induced by different scaffolds at week 4 after operation. a2) Masson's trichrome staining at week 4. a3) CD31 immunofluorescence staining of regenerated vessels at week 4. b) H&E, Masson's trichrome and CD31 staining of defect location in normal group. (Row 1: Overall observation of the cranial defect repair. The red arrow indicates the initial boundary of the defect. Row 2: Magnified view of the center and boundary site of the defects). (N: new bone tissue. S: scaffolds. V: new blood vessels (black arrow). F: fibrous tissue. B: old bone boundary). c1-4) Representative immunohistochemistry images of BMP-2, Runx2, OCN, VEGF at week 4 after implantation. d) Quantitative analysis of positive cells at week 4. (All scaffolds without cells. Blank: Defect alone with no treatment). (**p=0.0017, ***p=$5.2549\times10^{-6}$, **p=0.0005, *p=0.0322, *p=0.0192, ***p=$2.8877\times10^{-5}$, *p=0.0217, ***p=$6.9225\times10^{-7}$). (Two-sided comparison, Error bars represent standard deviation, *$p < 0.05$, **$p < 0.01$, and ***$p < 0.001$).

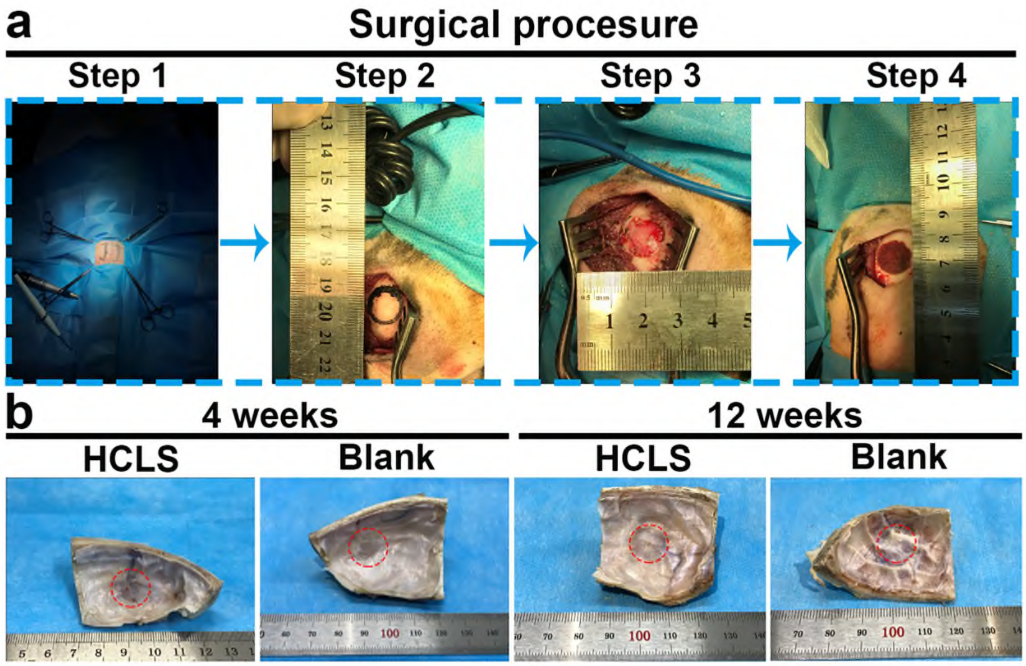


**Supplementary Figure 32.** *In situ* visual skull reconstruction effects by HCLS in beagle dog cranial defect model ($\Phi$ = 15 mm) at week 4 and 12. a) Schematic of cranioplasty in beagle dogs. b) Gross appearance of the regenerated tissue at 4 and 12 weeks of different treatments.

**Supplementary Table 1**. The primer sequences of Q-PCR for the osteogenesis-related genes expression test *in vitro*.

| Primer names | Sequences |
|---|---|
| Rab-RUNX2-F | 5′-TGGCGGGTAATGATGAAAAT-3′ |
| Rab-RUNX2-R | 5′-GAGGCGGTCAGAGAACAAA-3′ |
| Rab-OCN-F | 5′-GACACCATGAGGACCCTCTC-3′ |
| Rab-OCN-R | 5′-GCCTGGTAGTTGTTGTGAGC-3′ |
| Rab-OPN-F | 5′-CGCCGTGATTTGCTTTTGTC-3′ |
| Rab-OPN-R | 5′-GCATCCGGGTGTTTGTGGTA-3′ |
| Rab-Actin-F | 5′-TGGCTCTAACAGTCCGCCTAG-3′ |
| Rab-Actin-R | 5′-AGTGCGACGTGGACATCCG-3′ |

**Supplementary Table 2**. The primer sequences of Q-PCR test for the osteogenesis and angiogenesis-related genes expression of defect site in rabbit cranial defect model at week 12.

| Primer names | Sequences |
|---|---|
| Rab-RUNX2-F | GATGACGTCCCCGTCCATTC |
| Rab-RUNX2-R | GGAACAGGGTGGTGGAAGAC |
| Rab-VEGF-F | AGTTCGAGGAAAGGGCAAGG |
| Rab-VEGF-R | ACGCGAGTCTGTGTTTTTGC |
| Rab-BMP2-F | ACAAGTGGGAAAACCACCCG |
| Rab-BMP2-R | TGATGGAAACCGCTGTCGTC |
| Rab-OCN-F | CAGGCAGAGGCAAAGCCC |
| Rab-OCN-R | AGGGGATCCGGGTAAGGAG |
| Rab-OPN-F | TCACCACCATGAGAATCGCC |

| | |
|---|---|
| Rab-OPN-R | GCTTCTGAGATGGGTCAGGG |
| Rab-COL1-F | CGATGGCTTCCAGTTCGAGT |
| Rab-COL1-R | GCTACGCTGTTCTTGCAGTG |
| Rab-Actin-F | GAAACTCCGGCACGACAAAG |
| Rab-Actin-R | GATGATTCCCTCCCGGTGTC |
