# Supplementary material for: An instantly fixable and self-adaptive scaffold for skull regeneration by autologous stem cell recruitment and angiogenesis
Source: Nat Commun. 2022 May 6;13:2499. doi: 10.1038/s41467-022-30243-5 (PMC9076642; doi:10.1038/s41467-022-30243-5)
Supplement: Supplementary file 3 — Description to Additional Supplementary Information [file 41467_2022_30243_MOESM3_ESM.pdf]

Supplementary Figure 1. Characterization of micron hydroxyapatite.

Supplementary Figure 2. Synthesis and characterization of HAD.

Supplementary Figure 3. Full x-ray photoelectron spectroscopy spectra.

Supplementary Figure 4. Calcium ion chelating capacity analysis based on x-ray photoelectron spectroscopy spectra.

Supplementary Figure 5. Calcium chelation and mineralization of HCLS in SBF at 37°C for 7 days.

Supplementary Figure 6. Characterization of scaffolds.

Supplementary Figure 7. *In vitro* swelling test in Dulbecco's phosphate-buffered saline (DPBS) for DCLS, HCLS, HAD and Col I at 3, 7 and 14days.

Supplementary Figure 8. The original data of mechanical characterization of self-adhesive and flexible scaffold.

Supplementary Figure 9. Adhesion test of DCLS, HCLS, HAD and Col I on different substrates including glass, Ti, skull, pigskin and PE.

Supplementary Figure 10. SEM images of the interface between scaffold and host bone tissue on day 7 and 14.

Supplementary Figure 11. The original data of in vivo mechanical characterization of scaffold after implantation in rabbit skull for 7 and 14 days.

Supplementary Figure 12. Ca/P ratios and element analysis at the interface between implant and host bone based on EDS analysis after implanting in rabbit cranial defect.

Supplementary Figure 13. Response of scaffolds to macrophages *in vitro*.

Supplementary Figure 14. *In vivo* immune response of scaffold in mouse intramuscular and rabbit skull defect model.

Supplementary Figure 15. Flow cytometry data analysis.

Supplementary Figure 16. Representative immunostainings of CD197 and CD206, BMP-2 secretion and F4/80$^+$, and VEGF secretion and F4/80$^+$ macrophages in DCLS and HCLS on days 7 and 14 after implantation in mouse intramuscular.

Supplementary Figure 17. Representative local amplification immunostainings of CD197 and CD206, BMP-2 secretion and F4/80$^+$, as well as VEGF secretion and F4/80$^+$ macrophages in DCLS and HCLS on day 7 and 14 after intramuscular

implantation in mouse.

Supplementary Figure 18. Representative immunostainings of CD197 and CD206, BMP-2 secretion and F4/80$^+$, and VEGF secretion and F4/80$^+$ macrophages in DCLS and HCLS on days 7 and 14 after implantation in rabbit skull defect model.

Supplementary Figure 19. Representative local amplification immunostainings of CD197 and CD206, BMP-2 secretion and F4/80$^+$, and VEGF secretion and F4/80$^+$ macrophages in DCLS and HCLS on days 7 and 14 after implantation in rabbit skull defect model.

Supplementary Figure 20. Proliferation and morphology of BMSCs within DCLS, HCLS, HAD and Col I.

Supplementary Figure 21. Characterization of various specimens after 30 days subcutaneous implantation in nude mice.

Supplementary Figure 22. Immunofluorescence staining of Runx2 and CD31 after 30 days subcutaneous implantation in nude mice.

Supplementary Figure 23. *In vitro* BMSCs retention by DCLS, HCLS, HAD and Col I.

Supplementary Figure 24. *In Vitro* BMSCs recruitment by DCLS, HCLS, HAD and Col I.

Supplementary Figure 25. *In vivo* cell recruitment by DCLS, HCLS, HAD and Col I in a rabbit cranial defect model (Φ = 9 mm) after one week's implantation.

Supplementary Figure 26. CD90 immunofluorescence staining of BMSCs in scaffolds at one week after implantation in the rabbit cranial defect.

Supplementary Figure 27. Self-adhesive and flexible scaffold regulates gene expressions related to cellular activity, osteogenesis, angiogenesis, and ESCs recruitment on DCLS versus Col I.

Supplementary Figure 28. Self-adhesive and flexible scaffold regulates gene expressions related to cellular activity, osteogenesis, angiogenesis, and ESCs recruitment on HCLS versus DCLS.

Supplementary Figure 29. In situ visual skull reconstruction effects of DCLS and HCLS in rabbit cranial defect model (Φ = 9 mm).

Supplementary Figure 30. Characterization of various explants at week 4 and 12 after implantation in rabbit cranial defect model (Φ = 9 mm).

Supplementary Figure 31. *In situ* visual skull reconstruction effects by HCLS in beagle dog cranial defect model (Φ =15 mm) at week 4 and 12.

Supplementary Table 1. The primer sequences of Q-PCR for the osteogenesis-related genes expression test *in vitro*.

Supplementary Table 2. The primer sequences of Q-PCR test for the osteogenesis and angiogenesis-related genes expression of defect site in rabbit cranial defect model at week 12.

Supplementary Video 1. Instant fixability display of HCLS in wet environment.

Supplementary Video 2. Self-adaptivity display of HCLS.
