# Supplementary material for: An instantly fixable and self-adaptive scaffold for skull regeneration by autologous stem cell recruitment and angiogenesis
Source: Nat Commun. 2022 May 6;13:2499. doi: 10.1038/s41467-022-30243-5 (PMC9076642; doi:10.1038/s41467-022-30243-5)
Supplement: Supplementary file 6 — Reporting Summary [file 41467_2022_30243_MOESM6_ESM.pdf]

nature portfolio

Corresponding author(s): Yujiang Fan, Yong Sun

Last updated by author(s): Feb 24, 2022

Reporting Summary

Nature Portfolio wishes to improve the reproducibility of the work that we publish. This form provides structure for consistency and transparency in reporting. For further information on Nature Portfolio policies, see our Editorial Policies and the Editorial Policy Checklist.

Statistics

For all statistical analyses, confirm that the following items are present in the figure legend, table legend, main text, or Methods section.

n/a	Confirmed	
☐	☒	The exact sample size (*n*) for each experimental group/condition, given as a discrete number and unit of measurement
☐	☒	A statement on whether measurements were taken from distinct samples or whether the same sample was measured repeatedly
☐	☒	The statistical test(s) used AND whether they are one- or two-sided<br>*Only common tests should be described solely by name; describe more complex techniques in the Methods section.*
☐	☒	A description of all covariates tested
☐	☒	A description of any assumptions or corrections, such as tests of normality and adjustment for multiple comparisons
☐	☒	A full description of the statistical parameters including central tendency (e.g. means) or other basic estimates (e.g. regression coefficient) AND variation (e.g. standard deviation) or associated estimates of uncertainty (e.g. confidence intervals)
☐	☒	For null hypothesis testing, the test statistic (e.g. *F*, *t*, *r*) with confidence intervals, effect sizes, degrees of freedom and *P* value noted<br>*Give P values as exact values whenever suitable.*
☒	☐	For Bayesian analysis, information on the choice of priors and Markov chain Monte Carlo settings
☐	☒	For hierarchical and complex designs, identification of the appropriate level for tests and full reporting of outcomes
☐	☒	Estimates of effect sizes (e.g. Cohen's *d*, Pearson's *r*), indicating how they were calculated

Our web collection on statistics for biologists contains articles on many of the points above.

Software and code

Policy information about availability of computer code

Data collection	The MestReNova Version 10.0.1 and ChemDraw Version 18.0 softwares were used for the 1HNMR data analysis. X-ray photoelectron spectroscopy was conducted using Kratos AXIS ULTRA DLD photoelectron spectrometer. Images of immunostained cells were taken with a confocal laser scanning microscope (LSM 880, Zeiss) using ZEN software (Carl Zeiss Microscopy GmbH, Version 2.3.69.1000). Origin 8.0 and Graphpad Prism 7 were used for all other statistical analyses. The qRT-PCR data was collected using a CFX96 real-time PCR detection system (Bio-Rad CFX Manager 3.0). Commercial software Fiji ImageJ 1.8.0 was used to perform quantitative analysis of fluorescence intensity against background (Schindelin et al., 2012). Commercial software Mimics 17.0 was used to reconstruct microCT image. Image Lab software 5.2.1 was used for Western blotting analysis. Sections were scanned by an automatic digital slide scanner and analyzed by the Case Viewer 2.1 software (Pannoramic MIDI, 3D HISTECH, Hungary). All softwares used in this study have been described in detail in supplementary methods.
Data analysis	Commercial software flowJo (Version 10, FlowJo LLC) was used to analyze the results of flow cytometry. Statistical analyses were performed using Statistical software SPSS 22.0 and the results are reported as the mean±SD (standard deviation). Statistical differences were shown with three significance levels. * P<0.05, ** P<0.01 and *** P<0.001.

For manuscripts utilizing custom algorithms or software that are central to the research but not yet described in published literature, software must be made available to editors and reviewers. We strongly encourage code deposition in a community repository (e.g. GitHub). See the Nature Portfolio guidelines for submitting code & software for further information.

Data

Policy information about availability of data

All manuscripts must include a data availability statement. This statement should provide the following information, where applicable:

- Accession codes, unique identifiers, or web links for publicly available datasets
- A description of any restrictions on data availability
- For clinical datasets or third party data, please ensure that the statement adheres to our policy

The data that support the findings of this study are available within the article and its Supplementary Information files. The raw sequencing data generated in this study have been deposited in the NCBI SRA database under accession number PRJNA773733, and the BioProject's data is now publicly available at https://www.ncbi.nlm.nih.gov/bioproject/PRJNA773733. Genome Database (OryCun2.0_NCBI) and GO Database are used in the article and the corresponding links are listed as follows:ftp://ftp.ncbi.nlm.nih.gov/genomes/all/GCF/000/003/625/GCF_000003625.3_OryCun2.0/GCF_000003625.3_OryCun2.0_genomic.fna.gz and http://geneontology.org/. Other data are available from the authors upon reasonable request. The source data underlying Figure. 1-8 and Supplementary Figure 13, 14 and 30C are provided as a Source Data file. Source data are provided with this paper.

Field-specific reporting

Please select the one below that is the best fit for your research. If you are not sure, read the appropriate sections before making your selection.

☒ Life sciences ☐ Behavioural & social sciences ☐ Ecological, evolutionary & environmental sciences

For a reference copy of the document with all sections, see nature.com/documents/nr-reporting-summary-flat.pdf

Life sciences study design

All studies must disclose on these points even when the disclosure is negative.

Sample size	The sample sizes were estimated based on previous studies (C. Yu et al., J. Mater. Chem. B, 6, 5164-5173, 2018 and Y. Xu et al., Carbohydr. Polym. 250, 116979, 2020) and have been listed in the manuscript. For all in vitro studies, at least 3 random samples were selected for statistical analysis in each experiment based on the effect size and overlap between distributions. Sample size was selected for a minimum of n=3 as scaffolds generally show small variation in push-out test across samples. For in vivo cell recruitment studies, a minimum number of n=3 independent samples per group were utilized for observation of representative optical or confocal images to compare each groups. For all in vivo studies, at least 3 random samples were selected for statistical analysis in each experiment. The "n" number in the article represents several definitions, including biologically independent samples, independent experiments and cells examined over "n " independent experiments. Please refer to Author Checklist.
Data exclusions	No data points were excluded from analysis.
Replication	All experimental findings were reliably reproduced, and attempts at replication of experimental findings were successful. All, material preparation, processing and characterization were performed a minimum of 3 times. Both in vitro and in vivo experiments were replicated at least three times to confirm experimental trends prior to publication.
Randomization	Different treatments were assigned to animals randomly into control and experimental groups. For immunohistochemistry and immunofluorescence staining, the field of view was randomly selected for analysis. For western blot and RT-qPCR, samples were randomized and analyzed with standard approaches. Cell culture samples were assigned randomly, with control and experimental groups analysed in identical conditions to minimise potential covariates.
Blinding	For in vitro studies, blinding of test samples was not applicable since differences between groups were clear to the researchers by naked eye. However, the data were anonymized for statistical analysis. For in vivo studies, all investigators were blinded to group allocation during data collection. All assessments of functional recovery (new bone volume, osseointegration , etc.) were performed in a blinded manner. Immunohistochemical expression level was also performed blinded and the data were anonymized for statistical analysis.

Reporting for specific materials, systems and methods

We require information from authors about some types of materials, experimental systems and methods used in many studies. Here, indicate whether each material, system or method listed is relevant to your study. If you are not sure if a list item applies to your research, read the appropriate section before selecting a response.

Materials & experimental systems

n/a	Involved in the study
☐	☒ Antibodies
☐	☒ Eukaryotic cell lines
☒	☐ Palaeontology and archaeology
☐	☒ Animals and other organisms
☒	☐ Human research participants
☒	☐ Clinical data
☒	☐ Dual use research of concern

Methods

n/a	Involved in the study
☒	☐ ChIP-seq
☐	☒ Flow cytometry
☒	☐ MRI-based neuroimaging

Antibodies

Antibodies used

Anti-BMP-2 Receptor 1 (Abcam, ab6285, 1:100)
Anti-VEGF Receptor 1 (Abcam, ab32152, 1:250)
Goat anti-Mouse IgG (H+L) Cross-Adsorbed Secondary Antibody (Jackson, 115-545-003, 1:300)
Anti-Osteocalcin (Abcam, ab93876, 1:100)
Anti-Osteopontin (Abcam, ab166709, 1:200)
Anti-RUNX2 (Abcam, ab192256, 1:500)
Anti-collagen I Receptor 1 (NOVUS, NB600-450, 1:200)
goat anti-rabbit second antibody (Abcam, ab155079, 1:200)
goat anti-mouse second antibody (Abcam, ab150113, 1:400)
CD31 Monoclonal Antibody (JC/70A) (invitrogen, MA5-13188, 1:100)
Anti-CD31 (Abcam, ab9498, 1:250)
Goat anti-Rabbit IgG (H+L) Cross-Adsorbed Secondary Antibody, HRP (Thermo Fisher Scientific, G-21234, 1:100)
F4/80 (Abcam ab6640, 1:100, IF)
CD197 (Abcam, ab32527, 1:200, IF)
APC-conjugated CD197 (BioLegend, 12008, 1:80, FC)
CD206 (Abcam, ab8918, 1:100, IF)
VEGF (Abcam, ab52917, 1:250, IF)
FITC-conjugated CD206 (BioLegend, 141704, 1:100 FC)
Alexa Fluor 700-conjugated F4/80 (BioLegend, 123129, 1:200, FC)
CD90 (Abcam, ab181469, 1:200, IF)
CD44 Monoclonal Antibody (156-3C11) (invitrogen, MA5-13890, 1:100)
Goat anti-Mouse IgG (H+L) Highly Cross-Adsorbed Secondary Antibody, Alexa Fluor Plus 488 (Thermo Fisher Scientific, A32723, 1:100)
CD16 (eBioscience, H006T03B04, 1:100)
CD32 (eBioscience, 16-0329-38, 1:100)

Validation

Positive and negative controls of specific-binding (for each of the florescent labeled targets) were included in each experiment. Specific criteria are used to determine antibody eligibility for each application (ICC/IF, IHC, WB, flow cytometry, ELISA, IP, ChIP and polypeptide ChIP) by the manufacturer. We typically test each antibody in multiple repeated test for the same application. Antibody specificity is verified by a continuous gene knockout (KO) -validation program by the manufacturer. To ensure that different batches of the same antibody produced the same results, the manufacturer also assessed the differences between batches using conformance tests. Antibodies were used at the dilutions recommended by the manufacturer and the statements can be found on the manufactures' websites.

Eukaryotic cell lines

Policy information about cell lines

Cell line source(s)

The isolation of BMSCs was performed as previously described in our group (L Yuan et al., Tissue Eng. Part A., 2016). In brief, long bones with femoral heads but without cartilage were collected, and bone marrow was flushed using a syringe with alpha-modified Eagle's medium (a-MEM) containing 20% fetal bovine serum (FBS, GIBCO) and antibiotics (penicillin 100 U/mL, streptomycin 100 mg /mL). A cell strainer (70 mm, Falcon) was used to remove bone fragments. Filtered bone marrow cells were cultured in 10 cm cell culture dishes at 37 °C in a humidified atmosphere of 5% CO2. Nonadherent cells were removed by changing the medium after 24 h culture. The BMSCs were cultured and passaged until a confluence of 90% was achieved. The BMSCs of second passage were cultured in medium with 10% FBS and used in the following experiments. Mouse RAW 264.7 macrophages (Cell Bank of Chinese Academy of Sciences, Shanghai, China) were maintained in Dulbecco's modified Eagle's medium (DMEM; Gibco, USA) supplemented with 10% standard fetal bovine serum (FBS; Gibco, USA) and 1% penicillin/streptomycin (Gibco, USA).

Authentication

The BMSCs were authenticated by testing differentiation to a osteoblast phenotype with osteogenesis induction medium using an established protocol (Zuyuan Luo et al., Adv. Funct. Mater., 2018). Mouse RAW 264.7 macrophages were authenticated by testing inflammatory response to biphasic calcium phosphate ceramics and the resultant influence on osteogenic differentiation of mesenchymal stem cells using an established protocol (Jinjie Wu et al., J. Mater. Chem. B., 2021).

Mycoplasma contamination

We confirm that all cell lines were negative for mycoplasma contamination.

Commonly misidentified lines (See ICLAC register)	There was no commonly misidentified lines for our study.

Animals and other organisms

Policy information about studies involving animals; ARRIVE guidelines recommended for reporting animal research

Laboratory animals	BALB/cJGpt-Foxn1nu/Gpt nude mice (age about 28 days, around 16 g, male) were purchased from GemPharmatech Co., Ltd. (Nanjing, China) for ectopic osteogenesis test of materials. BALB/C mice (6 weeks, 16-20 g, male) and adult New Zealand white rabbits (2.5-3.0 kg, male, 2.5-3 months old) were purchased from the Laboratory Animal Center of Sichuan University (Chengdu, China) for in vivo inflammatory response test of materials. Adult male New Zealand white rabbits (2.5-3.0 kg, 2.5-3 months old) and adult male beagle dogs (6.7-8.5 kg, one year old) were purchased from Dossy Experimental Animals Co.,Ltd. (Chengdu, China) for skull reconstruction test of materials. All mice were housed in a specific pathogen-free environment and kept in a room with controlled temperature (~25 °C) and humidity under 12 h light/dark cycle. Animals were housed 1-2 per cage and maintained on a 12:12 light:dark cycle (lights on at 8 AM) with controlled room temperature (~25 °C) and humidity (50-80%); and given ad libitum access to laboratory chow and tap water throughout the study.
Wild animals	No wild animals were used in the study.
Field-collected samples	No field collected samples were used in the study.
Ethics oversight	All studies of in vivo inflammatory response test, ectopic osteogenesis and skull reconstruction were approved by the permission of the Sichuan University Ethics Committee (protocol number K2018001) and were carried out according to the institutional guidelines.

Note that full information on the approval of the study protocol must also be provided in the manuscript.

Flow Cytometry

Plots

Confirm that:

- [x] The axis labels state the marker and fluorochrome used (e.g. CD4-FITC).
- [x] The axis scales are clearly visible. Include numbers along axes only for bottom left plot of group (a 'group' is an analysis of identical markers).
- [x] All plots are contour plots with outliers or pseudocolor plots.
- [x] A numerical value for number of cells or percentage (with statistics) is provided.

Methodology

Sample preparation	Samples were dissected from euthanized mice after removing the surrounding muscle. To obtain a single-cell suspension of macrophages, the retrieved implants were isolated by trypsinization (Gibco) for 15 min at 37°C and washed with phosphate-buffered saline (PBS) three times. The resulting cell suspension was filtered (40 µm) and washed using a staining buffer. Subsequently, murine cells were blocked with CD16/CD32 monoclonal antibody (eBioscience) for 30 min at 4°C, and then immune cells were stained with Alexa Fluor 700-conjugated F4/80, APC-conjugated CD197, and FITC-conjugated CD206 for 45 min at 4°C. Appropriate isotypes were used, and ethidium monoazide bromide staining excluded dead cells.
Instrument	LSRFortessaTM X-20 flow cytometer system (BD Biosciences).
Software	The result of flow cytometry was analyzed by using flowJo (Version 10, FlowJo LLC).
Cell population abundance	FACS analysis was performed to a total cell number for at least 10^6 events per sample and acquisition of at least 10^5 viable and single events .
Gating strategy	The initial gating involved exclusion of debris with FSC/SSC by excluding low FSC/SSC values (extreme lower left quadrant), followed by positive selection for live cells, with a subsequent gate applied to select for macrophage cells while excluding other cells. The detailed gating strategy were seen in Supplementary Figures 15.

- [x] Tick this box to confirm that a figure exemplifying the gating strategy is provided in the Supplementary Information.
